# Predictions of Steady-State Photo-CIDNP Enhancement by Machine Learning

**DOI:** 10.1021/jacs.5c07462

**Published:** 2025-07-28

**Authors:** Marta Stefańska, Thomas Müntener, Sebastian Hiller

**Affiliations:** Biozentrum, 27209 University of Basel, Spitalstrasse 41, Basel 4056, Switzerland

## Abstract

Photochemically induced dynamic nuclear polarization (photo-CIDNP) is a hyperpolarization method used to boost signal sensitivity in NMR spectroscopy. So far, there is no theory to predict the steady-state photo-CIDNP enhancement reliably, and hence, suitable target molecules need to be identified through tedious experimental screenings. Here, we explore the use of machine learning to predict steady-state photo-CIDNP enhancement. For a series of 27 indole-, five amino-acid-, and eight phenol-derivatives, the signal-to-noise enhancement (SNE) of steady-state photo-CIDNP experiments was measured and then connected to a combination of eight molecular features. The nucleophilic Fukui index was identified as a strong qualitative indicator of the site with the highest SNE in each molecule. Furthermore, a semiquantitative machine learning model based on Logistic Regression identified the sites with high enhancements (SNE > 90) in 100% of cases. Among several quantitative machine learning models for enhancement prediction, CatBoost Regressor and K-Nearest Neighbors showed the best performance. The results demonstrate the high potential of machine learning approaches for predictions of photo-CIDNP SNE, which will enable virtual prescreening of compound libraries.

## Graphical abstract

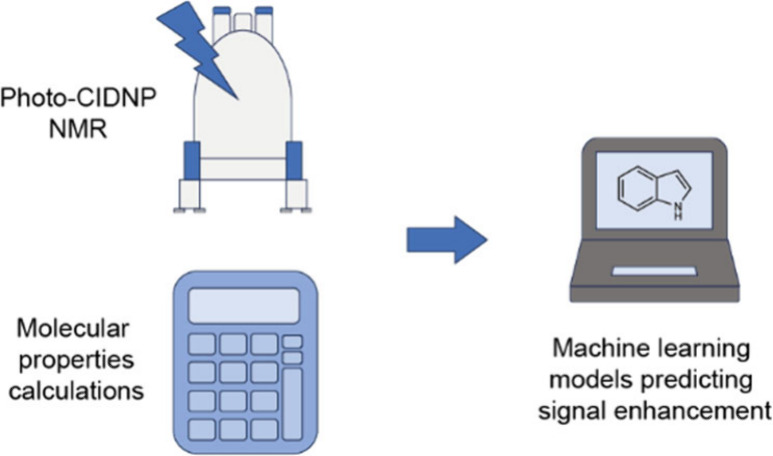

Hyperpolarization methods such as photochemically induced dynamic nuclear polarization (photo-CIDNP) can overcome the low sensitivity inherent to Nuclear Magnetic Resonance (NMR) spectroscopy.^1,2^ Photo-CIDNP has been applied in various contexts, including radical chemical reactions,^3,4^ hyperfine couplings determination,^5^ protein structures studies using photo-CIDNP active amino acids,^6–8^ ultrafast screening in drug development research,^9^ determination of the dissociation constants,^10^ and in magnetic resonance imaging (MRI).^11^

The two primary experiment types are time-resolved photo-CIDNP, i.e., with high laser power and short irradiation times, and steady-state photo-CIDNP, i.e., with low laser power but long irradiation times.^12^ Steady-state experiments require a cheaper and simpler light source with lower safety requirements, making them more available. Recent advancements in technology, such as new light sources,^13–15^ microfluidics systems,^16^ stable photosensitizers,^17,18^ and application to benchtop spectrometers,^19^ have raised interest in photo-CIDNP hyperpolarization. However, rationalizing the observed signal enhancement in steady-state photo-CIDNP is more difficult compared to time-resolved photo-CIDNP, in which polarization originates mostly from geminate pairs and can be directly correlated to hyperfine interactions.^12,20^ In steady-state experiments, the signal enhancement originates from multiple pathways, including geminate pairs, F-pairs, polarization transfer, and is also affected by relaxation during irradiation.^20^ It is important to note that not all molecules undergo photo-CIDNP hyperpolarization, and if they do, different targets, even with quite similar chemical structures, yield different enhancements.^21^ Consequently, suitable photo-CIDNP targets typically need to be identified through experimental screening, which is time- and resource-consuming and requires access to molecular libraries. It would thus be beneficial to use *in silico* methods to identify promising photo-CIDNP candidates prior to experimental screening.

Photo-CIDNP arises from the interaction between a photoexcited dye in singlet or triplet state and a target molecule.^1,20,22,23^ The underlying mechanism is composed of three steps: the interaction between the dye and the target molecule, the formation of the spin-correlated radical pair (SCRP) by electron transfer (ET), and finally, the α/β hyperpolarization caused by spin sorting. In certain cases, proton transfer precedes electron transfer toward a proton-coupled electron transfer (PCET) mechanism, typical for phenol-based molecules such as tyrosine.^24,25^ Each of the photo-CIDNP steps depends on various molecular properties, which thus have an influence on the final hyperpolarization. For time-resolved experiments, an analytical expression exists that relates the photo-CIDNP enhancement, which is in this case the geminate polarization Γ, to molecular properties:^20^

1 $$\Gamma \approx \frac{1}{12}\frac{\left|a_{iso}\right|}{\sqrt{2\left|g_{D}-g_{M}\right|\mu_{B}B_{0}/\hbar }}\sqrt{\frac{R^{2}}{D_{r}}}$$

where *a*_*iso*_ is the isotropic hyperfine coupling, *g*_*D*_ and *g*_*M*_ the g-factors of the dye and target molecule, *ℏ* the Planck constant, *R* the radical contact distance, *D*_*r*_ the diffusion coefficient, and *B*_*0*_ the magnetic field strength. For steady-state photo-CIDNP experiments, the underlying mechanism is more complicated, and consequently, there is currently no prediction of the enhancement based on molecular properties known.

In this work, we attempted to close this gap by establishing a quantitative correlation between the steady-state photo-CIDNP signal-to-noise enhancement (SNE) and selected molecular features. We recorded a complete data set on a compound library and connected the SNE data to molecular properties. We employed statistical analysis as well as semiquantitative and quantitative machine learning models to establish a prediction method for the steady-state photo-CIDNP effect.

Our compound library comprised a series of 27 indole-, 5 amino-acid-, and 8 phenol-derivatives as the target molecules (Table S1), with fluorescein used as the photoactive dye. We focused on indole and phenol cores with various substituents, because indole-based tryptophan and phenol-based tyrosine are among the most extensively studied photo-CIDNP molecules.^26–28^ For the entire library, we measured the site-specific SNE in steady-state photo-CIDNP ^1^H experiments at a magnetic field of 14.1 T (600 MHz proton Larmor frequency). For most indoles, one resonance showed a dominant enhancement over the others, and this resonance was usually proton 3 (Figure 1, Figure S1). The exception was the amino indole (N) series, which generally had low enhancement, likely due to their positive charge, which makes electron transfer more difficult and may quench the triplet state.^29,30^ For indoles containing hydroxyl groups, the highest enhancement was observed not for proton 3 but for a different one, depending on the substituent position, suggesting the PCET mechanism instead of ET. Surprisingly, compared to unsubstituted indole, the introduction of any substituent led to a lower enhancement. This likely occurs because substituents introduce steric hindrance and thus reduce favorable interactions with the dye. For amino-acid- and phenol-derivatives, the signal enhancements were significantly lower than for the indole-derivatives (Figure S2).

**Figure 1:**
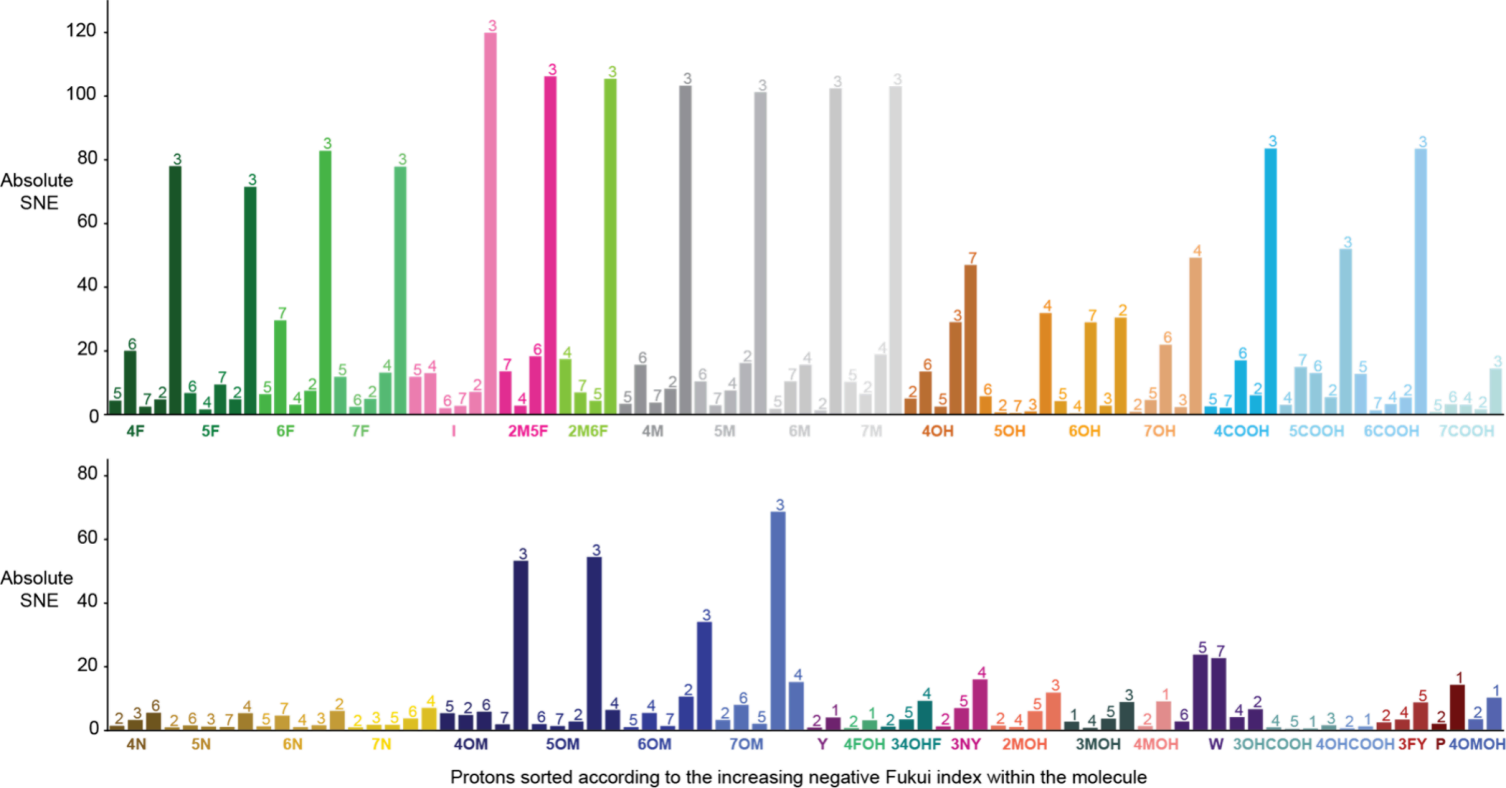
**The nucleophilic Fukui index is a good site-specific predictor of the steady-state photo-CIDNP effect.** Experimental photo-CIDNP effect for our compound library, given as absolute SNE. The abbreviations correspond to the individual molecules (Table S1), and numbers above the bars correspond to the specific protons (Figures S1–S2). For each molecule, the sites are sorted by increasing Fukui index. For 37 out of 40 tested molecules, the highest nucleophilic Fukui index corresponds to the highest enhancement within the molecule.

We selected eight molecular features that are likely influencing the steady-state photo-CIDNP effect and calculated them for each site in our compound library (Table S2): (1) *IP*, the ionization potential and (2) *N*, the nucleophilicity index. These correspond to the electron-donating properties from the molecule to the dye, which is a key step for radical pair formation. (3) LUMO–HOMO, the energy difference between the HOMO of the target molecule and the LUMO of the dye. This property plays an important role in the electron transfer. (4) *Δg*, the difference of *g*-factors between dye and molecule, and (5) *a*_*iso*_, the isotropic hyperfine interaction. These values are crucial for the triplet-singlet mixing, and both influence the geminate polarization, Γ. (6) *f*-, the nucleophilic Fukui index, which describes the electron density change upon electron removal^31^ and, thus, the probability of radical location. (7) *Q*, the probability of geminate polarization, given by

2 $$Q\approx \frac{1}{12}\frac{\left|a_{iso}\right|}{\sqrt{\frac{2\left|g_{D}-g_{M}\right|\mu_{B}B_{0}}{\hbar }}}$$

This expression was obtained by omitting the radical pair lifetime expression from eq 1 and assuming rapid diffusion beyond the exchange region.^20^ (8) *logP*, the partition coefficient of the hydrophobicity, as a measure for the overall hydrophobicity of the molecule. The interaction between molecules and dyes occurs via hydrophobic *π-π* stacking of the aromatic rings.^32^ For the molecules that contain hydroxyl substituents on aromatic rings, we calculated the *a*_*iso*_ *, g*, and *Q* values for both of the radicals formed via the ET and the PCET mechanism. Using Kaptein’s rules (see Methods), we then compared these values with the experimental observations to identify the underlying mechanism. The data showed that all such molecules were subject to the PCET mechanism, and the resulting radicals were then used in further analysis (Supporting Information Table S3).

To account for contributions to the hyperpolarization built-up from radical re-encounters (F-pairs), we also measured diffusion coefficients for all molecules by DOSY NMR. However, the diffusion coefficients were highly similar among the library and hence not further considered as a potential factor (Table S4).^33^ Furthermore, to account for partial relaxation in steady-state experiments, we measured proton *T*_*1*_ relaxation times (Table S5). For all protons, these were, however, longer than the laser irradiation time, thus excluding complete relaxation during the irradiation. We did not observe any correlation between SNEs and *T*_*1*_ for specific sites, so they were excluded from further analysis.

Among the eight features, the nucleophilic Fukui index stood out as a strong qualitative indicator of the position with the highest SNE within a molecule. For 37 of the 40 molecules, the position with the highest nucleophilic Fukui index had the largest photo-CIDNP SNE (Figure 1). This can be rationalized, because the Fukui index reflects the radical localization. A close proximity of unpaired electron to the nucleus leads to strong hyperfine interactions, causing larger triplet-singlet mixing rate differences between the α and β spin states, leading to a buildup of hyperpolarization.^34^ Accordingly, we also found a significant correlation between hyperfine interactions and nucleophilic Fukui indices (Figure S3), although hyperfine interactions alone were not strong indicators of the protons with the highest enhancement within the molecule (Figure S4). Moreover, at high magnetic fields, Zeeman interactions become stronger, making the influence of hyperfine interactions on photo-CIDNP significantly less pronounced.^20^ As possible alternative predictors of photo-CIDNP we also evaluated the electron density using Hirshfeld charges^35^ and Löwdin spin densities.^35^ Both parameters showed, however, weaker predictive performance than the nucleophilic Fukui index (Figures S5 and S6, Table S6). In particular, the direct correlation to the absolute SNEs was statistically less significant for the spin density (p-value = 2.35 × 10^–14^, Figure S6b) than for the nucleophilic Fukui index (p-value = 2.69 × 10^–19^, Figure S7h). This finding is readily rationalized, as the nucleophilic Fukui index captures a combination of molecular features relevant for the photo-CIDNP mechanism, such as the system reactivity, impact on the hyperfine interactions, electron donating properties, and radical localization. A detailed comparative discussion of these effects is included in Supporting Information.

We then assessed further correlations between the molecular features and the experimentally determined SNEs. Notably, the molecular properties have different coarse-graining. *logP* is a single value for all derivatives with the same substituent, irrespective of the substituent position. LUMO–HOMO, *IP, N*, and *Δg* are single values for each molecule, whereas *Q*, *f-, and a*_*iso*_ are specific to each proton in each molecule. We compared these values according to their hierarchy with the SNE values at three levels: across derivative families by taking into account the highest enhancement in the family; within molecules by taking the highest enhancement within the molecule; and for individual protons (Figure S7). At this coarse-graining, all the features show significance in relationship with SNEs (p-value <0.05, Figure S7–S10) – linear for most of them and quadratic for electron transfer-related properties (LUMO–HOMO, *IP*, and *N*), consistent with Marcus theory.^36^ To further investigate the correlations between photo-CIDNP activity and properties, we examined them in a reduced dimensional space, using Principal Component Analysis (PCA).^37^

The PCA analysis revealed a distinct region of highly enhanced (SNE > 90) and medium-enhanced (40 < SNE < 90) sites in molecular feature space, both in 2D and 3D PCA (Figure 2). Thereby, 2D PCA covered 70% of the variance in the data, and 3D covered 84% (Figure 2c). Hence, the sites with high photo-CIDNP activity have a distinct molecular feature combination. In the 3D PCA, PC1 strongly depends on the properties related to electron transfer, encapturing positive loadings from *IP* and LUMO–HOMO energy gap, and inverse loading from *N*. This feature set separates molecules by their electron-donating ability: weak donors have a high *IP*, a large HOMO–LUMO gap, and low *N*. Sites with high enhancement feature a narrow PC1 range (Figure S11), revealing a hidden relationship between features to drive photo-CIDNP activity together, proving that their combination is crucial for separating highly photo-CIDNP active sites. PC2 accounts for over 30% of additional variance and is dominated by the features characteristic for specific sites in molecules such as *f-, a*_*iso*_, and *Q*. PC3 is driven by loadings from the two remaining features–one molecular and one family-specific. Since these properties are shared across many sites, they contribute less to the overall variance. As observed from the PCA, combining multiple features appears beneficial for classification of SNEs. This conclusion is further supported by the ANOVA^38^ (Table S7) and Tukey’s^39^ (Table S8) tests, which identify statistically significant differences between groups. No single feature alone can fully explain the photo-CIDNP activity. Instead, the observed effects likely result from a complex interplay among the various molecular properties.

**Figure 2:**
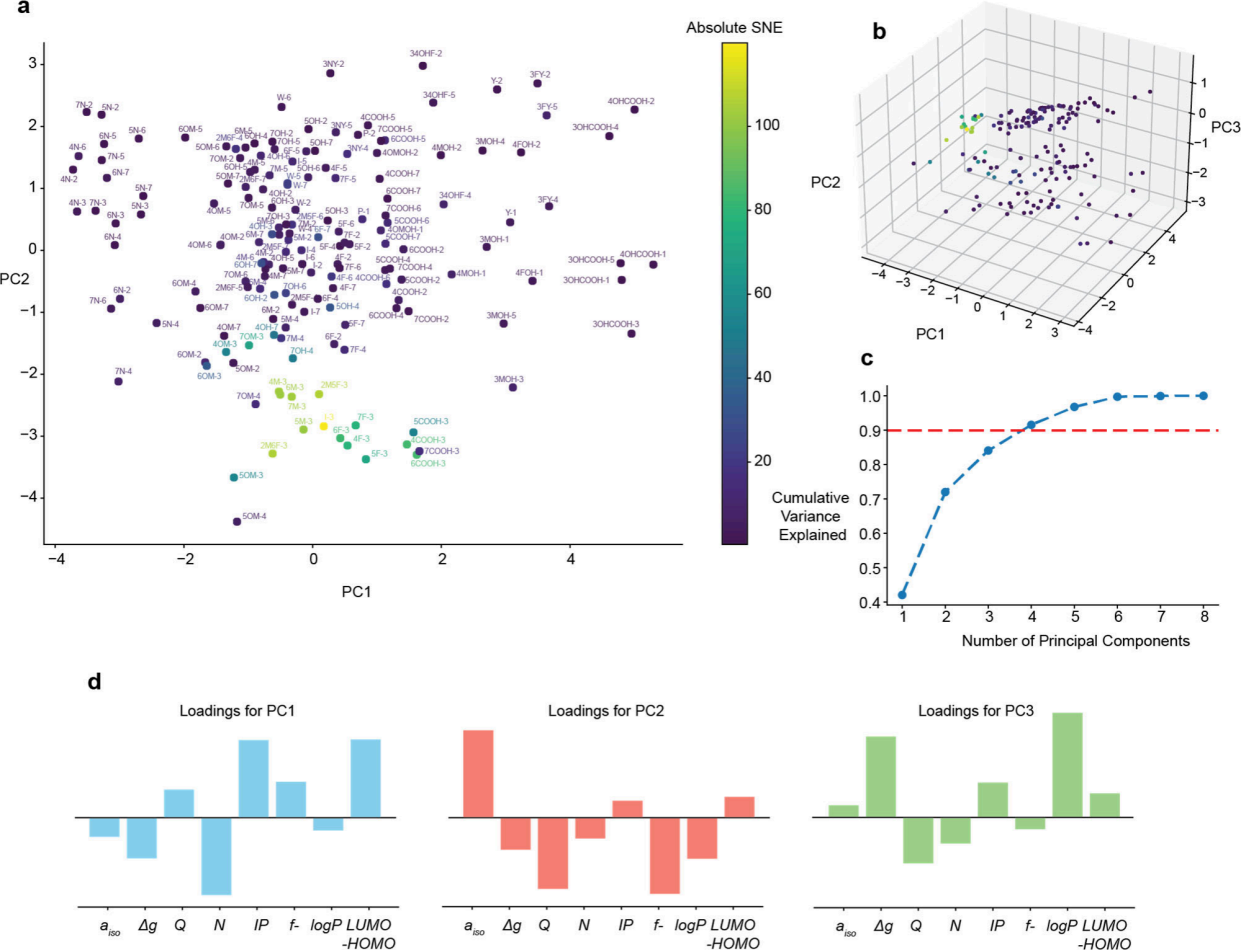
**Principal Component Analysis (PCA) effectively separates protons according to their photo-CIDNP enhancement.** (a) PCA across the first two principal components with assigned molecules and atoms (molecule code - atom number). The SNE is given in color code as indicated. (b) Same for three principal components. (c) Cumulative variance explained plot. (d) PCA loadings showing the features’ contributions to principal components, PC1-PC3.

Therefore, we explored the potential of machine learning to predict the absolute values of SNEs from the combined set of molecular properties. The first approach was done in a semiquantitative manner. The SNE of each proton was again classified as low (SNE < 40), medium (40 < SNE < 90), or high (SNE > 90) enhancement (Figure 3a). The SNE data was split into a training and a test set. Model based on logistic regression was trained and evaluated in a cumulative confusion matrix (Figure 3b). Since data split can impact model performance, especially in small data sets,^40^ we conducted one million independent runs with different random data splits. The diagonal elements of the confusion matrix correspond to the fraction of correct prediction of the enhancement category in all one million runs. The predictions accurately classified protons: 100% for high, 76% for medium, and 91% for low enhancement, making it a useful tool for identifying active molecules and excluding poor performers. Although the high enhancement category contains fewer protons (7) than the low enhancement category (143), the high accuracy of predictions suggests that sites with certain enhancement possess a unique combination of molecular properties identified by the model. Thereby, *logP*, *Δg*, and the site-specific Fukui index had the largest significance (Figure 3c), indicating that their interplay is most relevant to predict photo-CIDNP enhancement category.

**Figure 3:**
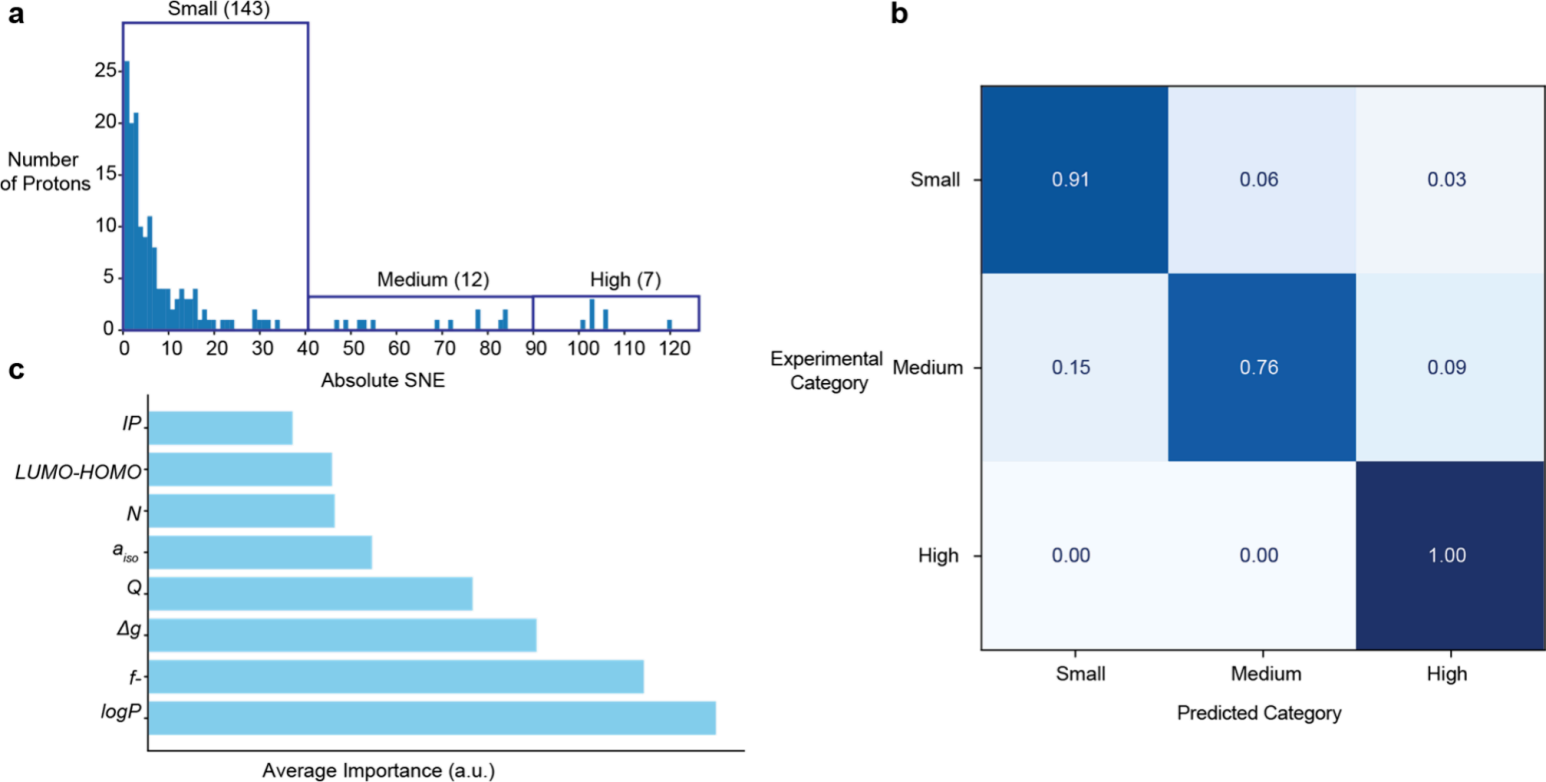
**Logistic regression can predict the photo-CIDNP enhancement class based on the molecular features.** (a) Protons classified into three categories based on their SNE. The number of protons in each category is shown in brackets. (b) Confusion matrix over one million iterations with different data splits. Diagonal cells indicate the proportion of correctly predicted samples. Off-diagonal cells represent misclassifications. (c) Feature importance averaged over one million runs.

To further refine the prediction, we developed a quantitative model to predict the exact SNE values for all protons. To this end, we tested a series of different machine-learning models. Each model was trained in 100 independent runs using a different data set into training, validation, and test sets. The performances of the individual models were evaluated by performance scores (see Methods). Among all models, CatBoost and K-Nearest Neighbors performed best (Figure 4a, Figure S12). The comparison between predicted and experimental SNEs for both models shows generally good correspondence (Figure 4b-c), with the largest errors being observed for indole. This is not surprising from a fundamental perspective, since indole has the highest enhancement, and the model cannot learn about it from the other molecules in the data set. Strikingly, the machine learning approach is mainly based on the nucleophilic Fukui index as the most impactful individual molecular feature (Figure 4d-e, Figure S13). This reconfirms our qualitative observation (Figure 1) in a quantitative fashion. Accordingly, the largest errors in machine learning prediction occur for those molecules where the qualitative correlation between the Fukui index and largest enhancement broke down (Figure 1).

**Figure 4:**
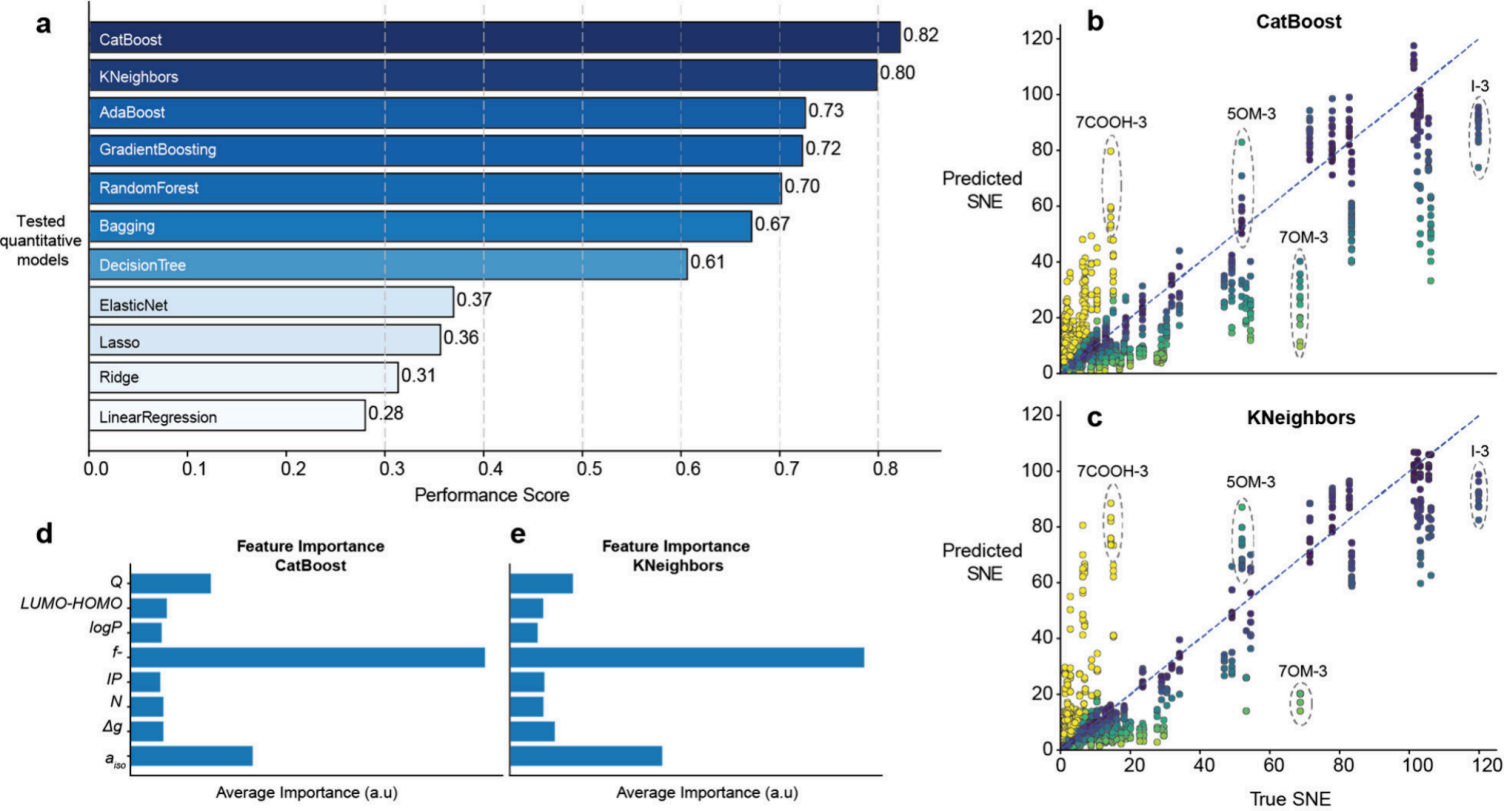
**CatBoost and KNeighbors models are the most efficient in quantitative predictions of SNEs based on molecular features.** (a) Performance scores for tested machine learning models calculated using over 100 iterations with different data splits. (b, c) Correlation between predicted and experimentally determined SNEs across 100 runs for CatBoost and KNeighbors models, respectively, with selected outliers marked and percentage errors scale. (d, e) Averaged feature importance across 100 runs for the best-performing models.

Overall, we have demonstrated in this work that both the class and magnitude of photo-CIDNP enhancement can be predicted using readily accessible molecular features for both PCET and ET mechanisms. Moreover, since the calculations are very fast, potential new targets can be efficiently evaluated considering both mechanisms. The high performance of the trained machine learning models is promising, given the limited size of our data set. With larger data sets, prediction performance is expected to improve further. The current data set can readily be extended in different directions. Different dyes could be incorporated by calculating corresponding *g* factors and LUMO energies, whereas a different strength of the magnetic fields could be included via the geminate polarization probability, *Q*. The results achieved in this work serve as a proof of concept, showing how machine learning models can identify promising photo-CIDNP candidates, thus accelerating molecule identification and reducing the need for extensive library screening.

## Supplementary Material



## Notes

Additional experimental details, materials, methods, statistical analysis of the model performance and the links to the GitHub with deposited source codes and input data are provided in Supporting Information with the remaining references (ref. [41–63]) (PDF)

## References

[1] Köckenberger, W. ; Matysik, J. Hyperpolarization Methods in NMR. In Encyclopedia of Spectroscopy and Spectrometry; Elsevier, 2017; pp 156–162.

[2] Lee J. H., Sekhar A., Cavagnero S. (2011). ^1^H-Detected ^13^C Photo-CIDNP as a Sensitivity Enhancement
Tool in Solution NMR. J. Am. Chem. Soc..

[3] Morozova O.
B., Korchak S. E., Vieth H.-M., Yurkovskaya A. V. (2009). Photo-CIDNP
Study of Transient Radicals of Met-Gly and Gly-Met Peptides in Aqueous
Solution at Variable pH. J. Phys. Chem. B.

[4] Troian-Gautier L., Mugeniwabagara E., Fusaro L., Cauet E., Kirsch-De
Mesmaeker A., Luhmer M. (2017). Photo-CIDNP Reveals Different Protonation
Sites Depending on the Primary Step of the Photoinduced Electron-/Proton-Transfer
Process with Ru­(II) Polyazaaromatic Complexes. J. Am. Chem. Soc..

[5] Pompe N., Illarionov B., Fischer M., Bacher A., Weber S. (2022). Completing
the Picture: Determination of ^13^C Hyperfine Coupling Constants
of Flavin Semiquinone Radicals by Photochemically Induced Dynamic
Nuclear Polarization Spectroscopy. J. Phys.
Chem. Lett..

[6] Kuhn L. T. (2013). Photo-CIDNP
NMR Spectroscopy of Amino Acids and Proteins. Top. Curr. Chem..

[7] Mok K. H., Hore P. J. (2004). Photo-CIDNP NMR Methods for Studying Protein Folding. Methods.

[8] Ding Y. H., Kiryutin A. S., Yurkovskaya A. V., Sosnovsky D. V., Sagdeev R. Z., Bannister S., Kottke T., Kar R. K., Schapiro I., Ivanov K. L., Matysik J. (2019). Nuclear Spin-Hyperpolarization
Generated in a Flavoprotein under Illumination: Experimental Field-Dependence
and Theoretical Level Crossing Analysis. Sci.
Rep..

[9] Torres F., Bütikofer M., Stadler G. R., Renn A., Kadavath H., Bobrovs R., Jaudzems K., Riek R. (2023). Ultrafast
Fragment
Screening Using Photo-Hyperpolarized (CIDNP) NMR. J. Am. Chem. Soc..

[10] Bütikofer M., Stadler G. R., Kadavath H., Cadalbert R., Torres F., Riek R. (2024). Rapid Protein-Ligand
Affinity Determination
by Photoinduced Hyperpolarized NMR. J. Am. Chem.
Soc..

[11] Bernarding J., Bruns C., Prediger I., Mützel M., Plau-mann M. (2024). Detection of Sub-Nmol Amounts of the Antiviral Drug
Favipiravir in 19F MRI Using Photo-Chemically Induced Dynamic Nuclear
Polarization. Sci. Rep..

[12] Morozova O. B., Ivanov K. L. (2019). Time-Resolved Chemically
Induced Dynamic Nuclear Polarization
of Biologically Important Molecules. ChemPhysChem.

[13] Bernarding J., Euchner F., Bruns C., Ringleb R., Müller D., Trantzschel T., Bargon J., Bommerich U., Plaumann M. (2018). Low-cost LED-based Photo-CIDNP Enables Biocompatible
Hyperpolarization of ^19^F for NMR and MRI at 7 and 4.7 T. ChemPhysChem.

[14] Yang H., Hofstetter H., Cavagnero S. (2019). Fast-Pulsing LED-Enhanced NMR: A
Convenient and Inexpensive Approach to Increase NMR Sensitivity. J. Chem. Phys..

[15] Bramham J. E., Golovanov A. P. (2022). Sample Illumination Device Facilitates in Situ Light-Coupled
NMR Spectroscopy without Fibre Optics. Commun.
Chem..

[16] Mompeán M., Sánchez-Donoso R. M., De La Hoz A., Saggiomo V., Velders A. H., Gomez M. V. (2018). Pushing
Nuclear
Magnetic Resonance Sensitivity Limits with Microfluidics and Photo-Chemically
Induced Dynamic Nuclear Polarization. Nat. Commun..

[17] Sobol, A. ; Torres, F. ; Aicher, A. ; Renn, A. ; Riek, R. Atto Thio 12 as a Promising Dye for Photo-CIDNP. J. Chem. Phys. 2019, 151 (23). 10.1063/1.5128575 31864237

[18] Okuno Y., Cavagnero S. (2016). Fluorescein:
A Photo-CIDNP Sensitizer Enabling Hypersensitive
NMR Data Collection in Liquids at Low Micromolar Concentration. J. Phys. Chem. B.

[19] Stadler G. R., Segawa T. F., Bütikofer M., Decker V., Loss S., Czarniecki B., Torres F., Riek R. (2023). Fragment Screening
and Fast Micromolar Detection on a Benchtop NMR Spectrometer Boosted
by Photoinduced Hyperpolarization. Angew. Chem.,
Int. Ed..

[20] Okuno Y., Cavagnero S. (2017). Photochemically Induced Dynamic Nuclear Polarization:
Basic Principles and Applications. eMagRes..

[21] Tada T., Shimajiri T., Nishimura K., Matsumoto N., Yanai N. (2025). Dye-quencher pair screening
for efficient photo-CIDNP: The role of
molecular diffusion. J. Chem. Phys..

[22] Goez M. (1995). An Introduction
to Chemically Induced Dynamic Nuclear Polarization. Concepts Magn. Reson..

[23] Goez M. (1997). Photochemically
Induced Dynamic Nuclear Polarization. Adv. Photochem..

[24] Costentin C., Louault C., Robert M., Savéant J.-M. (2009). The Electrochemical
Approach to Concerted Proton-Electron Transfers in the Oxidation of
Phenols in Water. Proc. Natl. Acad. Sci. U.S.A..

[25] Nilsen-Moe A., Reinhardt C. R., Glover S. D., Liang L., Hammes-Schiffer S., Hammarström L., Tommos C. (2020). Proton-Coupled Electron Transfer
from Tyrosine in the Interior of a de novo Protein: Mechanisms and
Primary Proton Acceptor. J. Am. Chem. Soc..

[26] Stob S., Kaptein R. (1989). Photo-CIDNP of the
Amino Acids. Photochem. Photobiol..

[27] Muszkat K. A., Wismontski-Knittel T. (1985). Reactivities
of Tyrosine, Histidine, Tryptophan, and
Methionine in Radical Pair Formation in Flavin Triplet Induced Protein
Nuclear Magnetic Polarization. Biochemistry.

[28] Ageeva A. A., Lukyanov R. S., Martyanova S. O., Magin I. M., Kruppa A. I., Polyakov N. E., Plyusnin V. F., Doktorov A. B., Leshina T. V. (2023). Photoinduced
Processes in Lysine-Tryptophan-Lysine Tripeptide with L and D Tryptophan. J. Mol. Sci..

[29] Fife D. J., Moore W. M. (1979). The Reduction and Quenching of Photoexcited Flavins
by EDTA. Photochem. Photobiol..

[30] Penzer, G. R. ; Radda, G. K. ; Taylor, J. A. ; Taylor, M. B. Chemical Properties of Flavins in Relation to Flavoprotein Catalysis. In Vitamins & Hormones; Harris, R. S. , Munson, P. L. , Diczfalusy, E. , Eds.; Academic Press, 1971; Vol. 28, pp 441–466. 10.1016/s0083-6729(08)60906-1 5004256

[31] Pucci R., Angilella G. G. N. (2022). Density Functional Theory, Chemical Reactivity, and
the Fukui Functions. Found Chem..

[32] Torres F., Renn A., Riek R. (2021). Exploration
of the Close Chemical
Space of Tryptophan and Tyrosine Reveals Importance of Hydrophobicity
in CW-Photo-CIDNP Performances. Magnetic Resonance.

[33] Kaptein R. (1971). Simple Rules
for Chemically Induced Dynamic Nuclear Polarization. J. Chem. Soc., Chem. Commun..

[34] Kawai A., Okutsu T., Obi K. (1991). Spin Polarization Generated in the
Triplet-Doublet Interaction: Hyperfine-Dependent Chemically Induced
Dynamic Electron Polarization. J. Phys. Chem..

[35] Lu T., Chen F. (2012). Comparison
of Computational Methods for Atomic Charges. Acta Phys. Chim. Sin..

[36] Marcus R. A. (1956). On the
Theory of Oxidation-Reduction Reactions Involving Electron Transfer. J. Chem. Phys..

[37] Jolliffe, I. T. Principal Component Analysis, 2nd ed.; Springer: New York, 2002.

[38] Fisher, R. A. The Design of Experiments; Oliver and Boyd: Edinburgh, 1935.

[39] Tukey, J. W. Exploratory Data Analysis; Addison-Wesley: Reading, MA, 1977.

[40] Safonova A., Ghazaryan G., Stiller S., Main-Knorn M., Nendel C., Ryo M. (2023). Ten Deep Learning Techniques to Address
Small Data Problems with Remote Sensing. Int.
J. Appl. Earth Obs. Geoinf..

