# Supplementary material for: Predictions of Steady-State Photo-CIDNP Enhancement by Machine Learning
Source: J Am Chem Soc. 2025 Jul 28;147(31):27172–8. doi: 10.1021/jacs.5c07462 (PMC12333361; doi:10.1021/jacs.5c07462)
Supplement: Supplementary file 1 [file ja5c07462_si_001.pdf]

Supporting information

Predictions of steady-state photo-CIDNP enhancement by machine learning

Marta Stefańska, Thomas Müntener, Sebastian Hiller*

This file contains:

- Materials and Methods
- Supplementary Tables S1–S8
- Supplementary Figures S1–S13
- References

Materials and methods

NMR spectroscopy

All indole derivatives and 3-fluoro-L-tyrosine were purchased from Fluorochem, 3,4-dihydroxy-L-phenylalanine and 4-methylphenol from Apollo, 3-amino-L-tyrosine from Cayman, 2-methylphenol and 3-methylphenol from Dr. Ehrenstorfer Reference Materials, tryptophan, tyrosine and fluorescein sodium salt from Sigma-Aldrich, and all the remaining compounds from BLD Pharm.

Stock solutions were prepared in DMSO-d_6 (indoles) or H_2O (others) in a concentration of 50 mM, then diluted to 5 mM. 50 mM stock solutions of tyrosine and its derivatives were prepared in 100 mM NaPi with pH=8.8 to improve solubility. All NMR samples were prepared in 100 mM phosphate buffer (pH=7.1) containing 25 μM of fluorescein, 100 μM of the target molecule, and 5% of D_2O. The NMR samples of the molecules from DMSO stock solutions, contained 2% DMSO in the final solution, which does not affect the photo-CIDNP enhancement. To prevent triplet state quenching by oxygen, an enzyme scavenging system was used, containing glucose oxidase (200 nM), catalase (140 nM), and D-glucose (2.5 mM)[41]. The sample volume for all the experiments was 500 μL.

All 1D ^{1}H NMR photo-CIDNP spectra were conducted on a 600 MHz Avance III spectrometer equipped with an inverse triple-resonance (TXI) room temperature probe at 298K. We used a modified perfect-echo W5 water suppression pulse sequence[42] with a laser irradiation block. The spectra were acquired with 4 dummy scans, 16 scans with an irradiation time of 1s, and a recovery delay of 3 s. The samples were irradiated using a laser diode emitting at 450 nm purchased from Thorlabs (L450P1600MM) with an output of 600 mW at the end of the optical fiber. The tip of the optical fiber was inserted into a 3 mm tube that was immersed in the 5 mm tube, touching the surface of the liquid. The 3 mm and 5 mm tubes were connected by a 3D-printed adapter.

The signal enhancement was evaluated by signal-to-noise enhancement (SNE), calculated according to the formula:

$$SNE = \frac{SN_{light}\sqrt{NS_{dark}}}{SN_{dark}\sqrt{NS_{light}}} \quad (1)$$

where SN corresponds to the signal-to-noise ratio, NS to the number of scans, light to irradiated spectrum, and dark to nonirradiated spectrum. The signal-to-noise ratios, SN, were calculated using the software TopSpin (Bruker). The measurement for each compound was repeated 3 times and the average SNE was taken as an input for the machine learning models.

All longitudinal relaxation times, T_1, and DOSY NMR spectra were recorded at 298K on a Bruker Avance-600 spectrometer equipped with a cryogenic triple-resonance probe. T_1 were measured with 4 dummy scans, 8 scans and 20 s of recycle delay using an inversion recovery experiment with excitation sculpting. DOSY experiments were measured with 4 dummy scans, 16 scans and 1 s of recycle delay, with stimulated echo using bipolar gradient pulses for diffusion and excitation sculpting for water suppression. Samples for T_1 and DOSY contained 1 mM of compounds and were prepared in 100 mM phosphate buffer (pH=7.1). T_1 and diffusion coefficients were calculated and extracted from the Dynamics Tool in TopSpin.

Theoretical calculations

Density functional theory (DFT) calculations were performed to optimize the molecular geometry using the B3LYP functional and 6-311++G(2d,2p) basis set[43] with incorporated Conductor-like Polarizable Continuum Model (CPCM) with a water solvent[44]. The calculations were performed in Orca 5.0[45]. The structure was optimized for both neutral molecules and cations to calculate the adiabatic ionization potential. The nucleophilicity indices, negative Fukui indices, Hirshfeld charges, HOMO, and LUMO energies were calculated on optimized structures by using Multiwfn software at B3LYP/6-31G* level[46,47]. The electronic structures to determine the g factors and the isotropic hyperfine interactions were calculated for the negatively charged fluorescein radical and positively charged radicals of the target molecules originating from ET using the EPR-II basis set[48]. Calculations for radicals derived from the PCET mechanism were performed on neutral structures lacking the hydroxyl hydrogen of the phenyl ring with the EPR-II basis set[48]. The *logP* indicating molecules hydrophobicity was predicted with the software DataWarrior[49]. The spin densities were calculated using unrestricted Kohn-Sham (UKS)[43] density functional theory at the B3LYP/6-311++G(2d,2p)[43] level, including solvation effects *via* the CPCM model (water as solvent)[44], calculated on geometrically optimized radicals originating form ET or PCET mechanism.

Kaptein's rule calculations

The expected sign of the photo-CIDNP-enhanced signals was calculated by Kaptein's rule[33] for net effect:

$$\Gamma = \mathrm{sgn}(\Delta g) \cdot \mathrm{sgn}(a_{iso}) \cdot \mu \cdot \varepsilon \tag{2}$$

and for multiplett effect[33]:

$$\Gamma = \mathrm{sgn}(a_{iso}(1)) \cdot \mathrm{sgn}(a_{iso}(2)) \cdot \mu \cdot \varepsilon \cdot J_{12} \cdot \sigma_{12} \tag{3}$$

, where $\Delta g = g_{Molecule} - g_{Dye}$, μ depends on the state of the initial radical pair (+1 for triplet, -1 for singlet precursor), and ε = +1 is for recombination products and ε = -1 for the escape products, J_{12} – the sign of J coupling between nuclei and σ_{12} is +1 when nuclei (1) and (2) belong to the same radical, and -1 when they belong to the different radicals. For the tested system, the initial radical pairs were formed in the triplet state (+1), as the triplet state of fluorescein has a sufficiently long lifetime to enable radical pair formation, unlike the short-lived excited singlet state[18]. This subsequently led to recombination products (+1). For the multiplett effect the σ_{12} = +1 as the nuclei belong to the same molecule. For the net effect +1 corresponds to the positive resonance, -1 to negative. For multiplett effect -1 corresponds to an absorptive/emissive lineshape (A/E), +1 to emissive/absorptive lineshape (E/A). The sign of the Kaptein rules was tested in Table S3 for radicals originating from PCET and ET

mechanism and then compared with the experimental sign to determine the photo-CIDNP mechanism.

Machine learning

All the codes were written in Python. The input data was loaded from an Excel file containing information about the molecular features and absolute values of SNE for all the protons from the tested molecules. We considered the absolute value of SNE as we are interested in the magnitude of the enhancement.

For logistic regression, the data was split into training (35 molecules, 87.5% of the data) and test sets (5 molecules, 12.5% of the data). Logistic regression models were implemented using the LogisticRegression class from the Scikit-learn library[50]. The models were configured to handle imbalanced classes. To ensure a robust evaluation, the models were trained and tested over one million iterations. In each iteration, a random shuffle of the molecules was performed to create new training and test sets. The features used for training were standardized using StandardScaler[50]. The training set was used in the learning process, and predictions were made on the test set. The model was updated across all iterations to accumulate feature importance values, which were computed based on the absolute values of the coefficients of the logistic regression model. The performance of the models was evaluated using the cumulative confusion matrix to assess the true positives, false positives, false negatives, and true negatives for each class (Small, Medium, High).

For quantitative models, the data was split into training (30 molecules, 75% of the data), validation (5 molecules, 12.5% of the data), and test sets (5 molecules, 12.5% of the data). The models tested in this study included the following from the scikit-learn library: Random Forest Regressor, Gradient Boosting Regressor, Linear Regression, K-Nearest Neighbors, Decision Tree Regressor, Lasso, Ridge, Elastic Net, Bagging Regressor and Ada Boost Regressor[51,52]. Additionally, we employed Cat Boost Regressor from the CatBoost library[53]. The process was iterated over different data splits 100 times to mitigate the potential impact of the data splitting on model performance[54]. The importance of features for different models was evaluated using various methods. For linear models (Logistic Regression, Linear Regression, Ridge, Elastic Net), feature importance was assessed based on coefficients assigned to each feature during training and validation. Tree-based models (Random Forest Regressor, Gradient Boosting Regressor, Decision Tree Regressor, Ada Boost Regressor, Cat Boost Regressor) used mean decrease in impurity (MDI), which was evaluated using mean squared error (MSE). For models that do not inherently provide feature importance or coefficients (K-Nearest Neighbors, Support Vector Regression, and Bagging Regressor), permutation importance was used.[55,56].

To evaluate quantitative models, we calculated statistical parameters for each iteration and then averaged them, including Coefficient of Determination, R^2, Mean Squared Error, MSE, Mean Absolute Percentage Error, MAPE, and Root Mean Squared Error, RMSE. The performance score, η, was calculated as:

$$\eta\,(100) = \frac{1}{100}\sum_{1}^{100} R^2 + \frac{1}{10^{-6} + \frac{1}{100}\sum_1^{100} MSE} + \frac{1}{10^{-6} + \frac{1}{100}\sum_1^{100} MAPE} + \frac{1}{10^{-6} + \frac{1}{100}\sum_1^{100} RMSE} \qquad \text{(S1)}$$

The 10^{-6} factor was used to prevent dividing by 0.
All source codes and the Excel input data file (Excel_input_file.xlsx) are available on GitHub: *https://github.com/hiller-lab/hyp_ml.git.*

Statistical analysis

All statistical analysis, including Pearson Correlation Coefficients, P-values, PCA, ANOVA and Tukey's test were performed in Python by using SciPy library[57].

Supplementary Discussion on Feature Scaling and Model Robustness

For enhanced robustness of the machine learning (ML) models, all quantum-chemical features were preprocessed using z-score normalization *via* the StandardScaler method from Scikit-learn library[50]. This procedure transforms each feature to have zero mean and unit variance across the training set, mitigating the effects of moderate systematic shifts introduced by variations in basis set. As a result, as long as each descriptor is computed consistently, *i.e.* using the same level of theory for both training and test datasets, the model's performance remains reliable. If a different level of theory is desired, the same ML architecture can be retrained on a newly generated dataset, assuming all descriptors are consistently computed at that level. The level of theory used must, of course, allow for reliable calculations without burdening the results with significant errors.

Geometry Optimization and Solvent Treatment

All molecules in this study were optimized at the B3LYP/6-311++G(2d,2p) level of theory, with solvent effects included *via* the conductor-like polarizable continuum model (CPCM). This setup is crucial, as changes in geometry and solvent modeling can significantly affect electronic structure and derived properties, especially site-sensitive descriptors such as Fukui indices and hyperfine coupling constants. These features are particularly important for ML model performance, as indicated by their high importance scores in both semi-quantitative and quantitative models. While alternative basis sets of similar size (*e.g.*, def2-TZVPD) produce only minor deviations in optimized geometries (< 0.01 Å in bond lengths, < 3 kJ mol^{-1} in relative energies), more substantial deviations from this reference level can affect sensitive properties, particularly hyperfine couplings, due to spin-density redistribution induced by small geometry changes. Therefore, consistency in geometry optimization and solvent treatment is critical to preserve model accuracy.

Sensitivity of Individual Descriptors to Basis Set Choice

1. **Nucleophilic Index (*N*)**

The nucleophilic index, defined as the HOMO energy of a molecule relative to that of a standard reference (e.g., TCNE), reflects its electron-donating ability[46,47]. Since *N* is HOMO-based, it exhibits moderate sensitivity to basis set selection, especially with

respect to the inclusion of diffuse functions. However, because *N* is computed as an energy difference, it is generally more robust than absolute orbital energies or Fukui indices. In our ML framework, *N* is z-standardized, and any consistent systematic bias introduced by the basis set is largely absorbed by this normalization.

2. **Ionization Potentials (IP)**

IPs are among the more robust quantum descriptors. Small Pople basis sets such as 6-31G tend to underestimate vertical or adiabatic IPs by ≈ 0.3–0.5 eV, whereas augmented basis sets (e.g., 6-31+G*, aug-cc-pVTZ) reduce this error to ≈ 0.1–0.3 eV. Composite methods such as CBS-QB3 or G4 typically achieve ≈ 0.1–0.2 eV accuracy[59]. Within our machine learning framework, consistent systematic biases introduced by the choice of basis set are effectively compensated by z-score normalization, thereby preserving model robustness across datasets computed at the same level of theory.

3. **HOMO–LUMO Gaps**

These are more sensitive to the choice of basis set, particularly the LUMO energy[60]. The feature should therefore be calculated with a level of theory not lower than B3LYP/6-31G* which can underestimate the LUMO energy but is consistent across data set.

4. **Fukui Indices**

Fukui indices are derived from orbital-resolved finite differences of electron density and thus inherit the sensitivity of both the frontier orbitals and the electron density to the basis set[61].

5. **Hyperfine Coupling Constants and g factors**

These properties are highly sensitive to the radial flexibility of the basis set, especially for hydrogen and heavy atoms. Replacing the specialized EPR-II basis set with a generic triple-ζ basis can alter isotropic couplings by up to ≈ 50%[62]. Δg-tensors, by contrast, are less affected[62]. Therefore, for future retraining of the model we recommend applying EPR II or EPR III.

Additional Comment on the Chosen Level of Theory

The application of B3LYP/6-31G* is well justified as has it been used to generate electronic structures for approximately 86 million molecules, creating a publicly accessible dataset widely adopted in ML-based chemical modeling[63]. Moreover, B3LYP/6-31G* is the default level of theory in Multiwfn[46,47], the software used for some features calculation in this study.

Nucleophilic Fukui Indices as descriptors of photo-CIDNP enhancement

In our study, we identified the nucleophilic Fukui index as the best single parameter to predict the local site of maximal photo-CIDNP signal enhancement and as a key contributing parameter for predictions of the exact value of the enhancements (SNE) across a molecular dataset. The nucleophilic Fukui index is defined as the change in electron density upon removal of an electron from the neutral molecule (*i.e.,* $f^- = \rho_N - \rho_{N-1}$). It therefore identifies

electron-rich, donor-prone sites, and these are precisely the locations most susceptible to radical formation upon electron donation to the dye, which in turn is the core mechanism of photo-CIDNP. Photo-CIDNP hyperpolarization arises from the formation of spin-correlated radical pairs (SCRPs) through electron transfer process between the target molecule and the dye, the regions with the highest nucleophilic Fukui indices are expected to be the most reactive toward such processes. These reactive sites are directly involved in the radical localization and therefore strongly influence the resultant hyperfine interactions, which govern the spin dynamics underlying the photo-CIDNP signal enhancement. In comparison, spin density maps alone offer insight into the localization of the unpaired electron in the radical state, but do not necessarily reflect the molecule's intrinsic tendency to form a radical or engage in electron transfer process. The nucleophilic Fukui index is therefore a better predictor than spin density or electron density alone.

Supplementary Tables

Table S1. Composition of the compound library used in this study.

Molecule	Abbreviation	Molecule	Abbreviation
2-methyl-5-fluoroindole	2M5F	Tyrosine	Y
2-methyl-6-fluoroindole	2M6F	4-Fluorophenol	4FOH
Indole	I	3,4-Dihydroxy-L-phenylalanine	34OHF
4-fluoroindole	4F	3-Amino-L-tyrosine	3NY
5-fluoroindole	5F	2-Methylphenol	2MOH
6-fluoroindole	6F	3-Methylphenol	3MOH
7-fluoroindole	7F	4-Methylphenol	4MOH
4-methylindole	4M	3-Hydroxybenzoic acid	3OHCOOH
5-methylindole	5M	4-Hydroxybenzoic acid	4OHCOOH
6-methylindole	6M	4-Methoxyphenol	4OMOH
7-methylindole	7M	Tryptophan	W
4-hydroxyindole	4OH	3-Fluoro-L-tyrosine	3FY
5-hydroxyindole	5OH	Paracetamol	P
6-hydroxyindole	6OH		
7-hydroxyindole	7OH		
Indole-4-carboxylic acid	4COOH		
Indole-5-carboxylic acid	5COOH		
Indole-6-carboxylic acid	6COOH		
Indole-7-carboxylic acid	7COOH		
4-aminoindole	4N		
5-aminoindole	5N		
6-aminoindole	6N		
7-aminoindole	7N		
4-methoxyindole	4OM		
5-methoxyindole	5OM		
6-methoxyindole	6OM		
7-methoxyindole	7OM		

Table S2. Molecular features of all protons in the compound library, along with the experimental photo-CIDNP SNE.

Molecule	Atom	Absolute SNE	a_{iso} (MHz)	Δg (a.u.)	Nucleophilicity index, N (a.u.)	Adiabatic ionization potential, IP (eV)	Negative Fukui index, f-	logP (a.u.)	Geminate polarization, Q (a.u.)	LUMO-HOMO (Hartree)
2M5F	3	106.2	-17.67	0.000662	3.3612	5.41924	0.0561	2.1977	3.64E+01	5.697165
2M5F	4	2.8	-17.95	0.000662	3.3612	5.41924	0.0356	2.1977	3.69E+01	5.697165
2M5F	6	18.3	-4.67	0.000662	3.3612	5.41924	0.0358	2.1977	9.61E+00	5.697165
2M5F	7	13.5	-12.66	0.000662	3.3612	5.41924	0.031	2.1977	2.60E+01	5.697165
I	2	7.1	-7.99	0.0005839	3.3574	5.44300	0.0436	1.699	1.75E+01	5.700965
I	3	119.9	-25.76	0.0005839	3.3574	5.44300	0.0532	1.699	5.64E+01	5.700965
I	7	2.7	-17.98	0.0005839	3.3574	5.44300	0.0384	1.699	3.94E+01	5.700965
I	5	11.8	1.63	0.0005839	3.3574	5.44300	0.0303	1.699	3.57E+00	5.700965
I	6	2.0	-11.41	0.0005839	3.3574	5.44300	0.0369	1.699	2.50E+01	5.700965
I	4	12.9	-9.63	0.0005839	3.3574	5.44300	0.0363	1.699	2.11E+01	5.700965
4F	2	4.8	-6.83	0.0002465	3.2784	5.52736	0.0416	1.7998	2.30E+01	5.779965
4F	3	78.0	-24.85	0.0002465	3.2784	5.52736	0.0514	1.7998	8.38E+01	5.779965
4F	5	4.4	0.02	0.0002465	3.2784	5.52736	0.0317	1.7998	6.74E-02	5.779965
4F	6	20.0	-10.83	0.0002465	3.2784	5.52736	0.0375	1.7998	3.65E+01	5.779965
4F	7	2.5	-11.66	0.0002465	3.2784	5.52736	0.0387	1.7998	3.93E+01	5.779965
5F	2	4.8	-4.25	0.0004991	3.2272	5.58528	0.0419	1.7998	1.01E+01	5.831165
5F	3	71.5	-28.65	0.0004991	3.2272	5.58528	0.0548	1.7998	6.79E+01	5.831165
5F	7	9.4	-18.80	0.0004991	3.2272	5.58528	0.0389	1.7998	4.45E+01	5.831165
5F	6	6.7	-7.88	0.0004991	3.2272	5.58528	0.0338	1.7998	1.87E+01	5.831165
5F	4	1.6	-9.42	0.0004991	3.2272	5.58528	0.0348	1.7998	2.23E+01	5.831165
6F	2	6.9	-14.24	0.0003024	3.305	5.47757	0.048	1.7998	4.33E+01	5.753365
6F	3	82.8	-23.83	0.0003024	3.305	5.47757	0.0535	1.7998	7.25E+01	5.753365
6F	4	3.0	-15.54	0.0003024	3.305	5.47757	0.0357	1.7998	4.73E+01	5.753365
6F	5	6.4	4.40	0.0003024	3.305	5.47757	0.028	1.7998	1.34E+01	5.753365
6F	7	29.6	-7.36	0.0003024	3.305	5.47757	0.0352	1.7998	2.24E+01	5.753365
7F	2	4.9	-4.98	0.0003475	3.2486	5.57062	0.0403	1.7998	1.41E+01	5.809765
7F	3	77.9	-24.82	0.0003475	3.2486	5.57062	0.0501	1.7998	7.05E+01	5.809765
7F	4	13.2	-19.63	0.0003475	3.2486	5.57062	0.0413	1.7998	5.57E+01	5.809765
7F	5	11.8	-0.36	0.0003475	3.2486	5.57062	0.0331	1.7998	1.01E+00	5.809765
7F	6	2.4	-10.77	0.0003475	3.2486	5.57062	0.0369	1.7998	3.06E+01	5.809765
4M	2	8.1	-7.75	0.0005844	3.4807	5.32431	0.0412	2.0429	1.70E+01	5.577665
4M	3	103.2	-22.66	0.0005844	3.4807	5.32431	0.0488	2.0429	4.96E+01	5.577665
4M	5	3.3	-0.28	0.0005844	3.4807	5.32431	0.0304	2.0429	6.20E-01	5.577665
4M	6	15.6	-10.20	0.0005844	3.4807	5.32431	0.0372	2.0429	2.23E+01	5.577665
4M	7	3.7	-11.82	0.0005844	3.4807	5.32431	0.0381	2.0429	2.59E+01	5.577665
5M	2	16.1	-4.15	0.0005561	3.4143	5.39129	0.0406	2.0429	9.31E+00	5.644065
5M	3	101.3	-26.08	0.0005561	3.4143	5.39129	0.0521	2.0429	5.85E+01	5.644065
5M	4	7.5	-19.45	0.0005561	3.4143	5.39129	0.0386	2.0429	4.36E+01	5.644065
5M	6	10.4	-7.27	0.0005561	3.4143	5.39129	0.0334	2.0429	1.63E+01	5.644065
5M	7	2.9	-11.55	0.0005561	3.4143	5.39129	0.0365	2.0429	2.59E+01	5.644065
6M	2	1.3	-13.34	0.0006084	3.4791	5.30804	0.0457	2.0429	2.86E+01	5.579265
6M	3	102.4	-21.73	0.0006084	3.4791	5.30804	0.051	2.0429	4.66E+01	5.579265
6M	4	15.6	-15.57	0.0006084	3.4791	5.30804	0.0358	2.0429	3.34E+01	5.579265
6M	5	1.8	4.42	0.0006084	3.4791	5.30804	0.0269	2.0429	9.49E+00	5.579265
6M	7	10.4	-8.64	0.0006084	3.4791	5.30804	0.035	2.0429	1.85E+01	5.579265
7M	2	6.4	-4.35	0.0005668	3.4407	5.37229	0.0392	2.0429	9.66E+00	5.617665
7M	3	103.1	-23.39	0.0005668	3.4407	5.37229	0.0487	2.0429	5.20E+01	5.617665
7M	4	18.8	-19.73	0.0005668	3.4407	5.37229	0.0407	2.0429	4.39E+01	5.617665
4OH	2	5.0	-5.09	-0.0010279	3.6131	5.19125	0.0356	1.3533	8.40E+00	5.445265
4OH	3	27.7	-7.75	-0.0010279	3.6131	5.19125	0.0423	1.3533	1.28E+01	5.445265
4OH	5	2.0	-18.47	-0.0010279	3.6131	5.19125	0.0366	1.3533	3.05E+01	5.445265
4OH	6	11.2	4.35	-0.0010279	3.6131	5.19125	0.0361	1.3533	7.19E+00	5.445265
4OH	7	47.0	-25.38	-0.0010279	3.6131	5.19125	0.0427	1.3533	4.19E+01	5.445265
5OH	2	0.8	1.12	-0.0016573	3.478	5.32240	0.0304	1.3533	1.46E+00	5.580365
5OH	3	1.0	-5.48	-0.0016573	3.478	5.32240	0.044	1.3533	7.13E+00	5.580365
5OH	4	31.9	-22.03	-0.0016573	3.478	5.32240	0.0442	1.3533	2.86E+01	5.580365
5OH	6	6.2	-10.27	-0.0016573	3.478	5.32240	0.0287	1.3533	1.33E+01	5.580365
5OH	7	0.5	1.57	-0.0016573	3.478	5.32240	0.0355	1.3533	2.04E+00	5.580365
6OH	2	30.5	-16.74	-0.0012988	3.6355	5.13497	0.0462	1.3533	2.46E+01	5.422865
6OH	3	2.8	-0.98	-0.0012988	3.6355	5.13497	0.0457	1.3533	1.44E+00	5.422865
6OH	4	0.4	1.66	-0.0012988	3.6355	5.13497	0.0332	1.3533	2.44E+00	5.422865
6OH	5	4.2	-8.59	-0.0012988	3.6355	5.13497	0.0285	1.3533	1.26E+01	5.422865
6OH	7	29.0	-19.12	-0.0012988	3.6355	5.13497	0.0362	1.3533	2.81E+01	5.422865
7OH	2	0.9	4.06	-0.0010661	3.5664	5.24744	0.0329	1.3533	6.59E+00	5.491965
7OH	3	2.5	-6.93	-0.0010661	3.5664	5.24744	0.0412	1.3533	1.12E+01	5.491965

7OH	4	49.2	-27.96	-0.0010661	3.5664	5.24744	0.0449	1.3533	4.53E+01	5.491965
7OH	5	4.9	4.35	-0.0010661	3.5664	5.24744	0.0347	1.3533	7.05E+00	5.491965
7OH	6	23.6	-20.10	-0.0010661	3.5664	5.24744	0.0401	1.3533	3.26E+01	5.491965
4COOH	2	6.0	-11.36	0.0003806	3.0937	5.69894	0.0453	1.1841	3.08E+01	5.964665
4COOH	3	83.5	-27.71	0.0003806	3.0937	5.69894	0.0525	1.1841	7.52E+01	5.964665
4COOH	5	2.5	5.12	0.0003806	3.0937	5.69894	0.0241	1.1841	1.39E+01	5.964665
4COOH	6	17.0	-14.91	0.0003806	3.0937	5.69894	0.0362	1.1841	4.05E+01	5.964665
4COOH	7	2.1	-3.36	0.0003806	3.0937	5.69894	0.0304	1.1841	9.13E+00	5.964665
5COOH	2	5.5	-9.32	0.0005553	3.0646	5.71038	0.0445	1.1841	2.09E+01	5.993765
5COOH	3	52.0	-27.09	0.0005553	3.0646	5.71038	0.0536	1.1841	6.08E+01	5.993765
5COOH	4	3.0	-16.56	0.0005553	3.0646	5.71038	0.0321	1.1841	3.72E+01	5.993765
5COOH	7	14.9	-12.24	0.0005553	3.0646	5.71038	0.0323	1.1841	2.75E+01	5.993765
5COOH	6	13.0	-7.79	0.0005553	3.0646	5.71038	0.0339	1.1841	1.75E+01	5.993765
6COOH	2	5.3	-6.40	0.0003365	3.0666	5.73552	0.0413	1.1841	1.85E+01	5.991765
6COOH	3	83.5	-28.08	0.0003365	3.0666	5.73552	0.053	1.1841	8.10E+01	5.991765
6COOH	4	3.2	-17.56	0.0003365	3.0666	5.73552	0.0368	1.1841	5.07E+01	5.991765
6COOH	5	12.7	0.48	0.0003365	3.0666	5.73552	0.027	1.1841	1.38E+00	5.991765
6COOH	7	1.3	-6.85	0.0003365	3.0666	5.73552	0.0292	1.1841	1.98E+01	5.991765
7COOH	2	1.6	-12.02	0.0004761	3.0569	5.71307	0.0474	1.1841	2.92E+01	6.001465
7COOH	3	14.4	-27.59	0.0004761	3.0569	5.71307	0.056	1.1841	6.69E+01	6.001465
7COOH	4	3.1	-15.12	0.0004761	3.0569	5.71307	0.0333	1.1841	3.67E+01	6.001465
7COOH	5	0.8	1.81	0.0004761	3.0569	5.71307	0.0271	1.1841	4.38E+00	6.001465
7COOH	6	3.2	-9.30	0.0004761	3.0569	5.71307	0.0299	1.1841	2.26E+01	6.001465
4N	2	1.4	-4.85	0.0004341	4.1217	4.67753	0.0286	1.0217	1.23E+01	4.936665
4N	3	3.3	-9.00	0.0004341	4.1217	4.67753	0.0318	1.0217	2.29E+01	4.936665
4N	6	5.5	0.53	0.0004341	4.1217	4.67753	0.0329	1.0217	1.35E+00	4.936665
5N	2	0.9	3.96	0.0003861	3.8338	4.75127	0.0239	1.0217	1.07E+01	4.936665
5N	3	1.2	-10.08	0.0003861	3.8338	4.75127	0.0303	1.0217	2.71E+01	5.224565
5N	4	5.4	-17.94	0.0003861	3.8338	4.75127	0.0426	1.0217	4.83E+01	5.224565
5N	6	1.5	-0.44	0.0003861	3.8338	4.75127	0.0281	1.0217	1.18E+00	5.224565
5N	7	1.1	-6.41	0.0003861	3.8338	4.75127	0.0318	1.0217	1.73E+01	5.224565
6N	2	6.1	-16.66	0.0004923	3.9553	4.67009	0.0404	1.0217	3.97E+01	5.103065
6N	3	1.6	-7.43	0.0004923	3.9553	4.67009	0.0369	1.0217	1.77E+01	5.103065
6N	7	3.6	-5.00	0.0004923	3.9553	4.67009	0.0302	1.0217	1.19E+01	5.103065
6N	5	1.3	-1.28	0.0004923	3.9553	4.67009	0.028	1.0217	3.06E+00	5.103065
6N	4	1.0	-11.18	0.0004923	3.9553	4.67009	0.0363	1.0217	2.67E+01	5.103065
7N	2	0.9	3.55	0.0004042	4.0464	4.75104	0.0233	1.0217	9.35E+00	5.011965
7N	3	1.7	-10.73	0.0004042	4.0464	4.75104	0.0287	1.0217	2.82E+01	5.011965
7N	4	7.1	-24.11	0.0004042	4.0464	4.75104	0.0467	1.0217	6.35E+01	5.011965
7N	5	1.8	1.88	0.0004042	4.0464	4.75104	0.0339	1.0217	4.95E+00	5.011965
7N	6	3.7	-17.24	0.0004042	4.0464	4.75104	0.0408	1.0217	4.54E+01	5.011965
4OM	2	4.8	-7.35	0.0001691	3.6543	5.13645	0.0366	1.629	2.99E+01	5.404065
4OM	3	53.3	-16.65	0.0001691	3.6543	5.13645	0.0429	1.629	6.78E+01	5.404065
4OM	5	5.4	-5.22	0.0001691	3.6543	5.13645	0.0313	1.629	2.12E+01	5.404065
4OM	6	5.9	-7.18	0.0001691	3.6543	5.13645	0.0368	1.629	2.92E+01	5.404065
4OM	7	1.9	-15.63	0.0001691	3.6543	5.13645	0.0412	1.629	6.36E+01	5.404065
5OM	7	1.4	5.40	-3.51E-05	3.5917	5.21686	0.0288	1.629	4.82E+01	5.466665
5OM	3	54.5	-19.45	-3.51E-05	3.5917	5.21686	0.0411	1.629	1.74E+02	5.466665
5OM	4	6.4	-21.37	-3.51E-05	3.5917	5.21686	0.0449	1.629	1.91E+02	5.466665
5OM	6	2.0	2.84	-3.51E-05	3.5917	5.21686	0.0244	1.629	2.54E+01	5.466665
5OM	2	2.8	-13.02	-3.51E-05	3.5917	5.21686	0.0359	1.629	1.16E+02	5.466665
6OM	2	10.6	-14.68	8.48E-05	3.7151	5.05950	0.0428	1.629	8.44E+01	5.343265
6OM	3	34.1	-14.55	8.48E-05	3.7151	5.05950	0.0439	1.629	8.37E+01	5.343265
6OM	4	5.5	-11.56	8.48E-05	3.7151	5.05950	0.0342	1.629	6.65E+01	5.343265
6OM	5	1.0	3.09	8.48E-05	3.7151	5.05950	0.0246	1.629	1.77E+01	5.343265
6OM	7	1.4	-11.56	8.48E-05	3.7151	5.05950	0.0379	1.629	6.65E+01	5.343265
7OM	2	3.3	1.80	0.0001868	3.5677	5.24621	0.0304	1.629	6.96E+00	5.490665
7OM	3	68.7	-18.13	0.0001868	3.5677	5.24621	0.0393	1.629	7.02E+01	5.490665
7OM	4	15.2	-22.19	0.0001868	3.5677	5.24621	0.045	1.629	8.59E+01	5.490665
7OM	5	2.1	-2.01	0.0001868	3.5677	5.24621	0.036	1.629	7.78E+00	5.490665
7OM	6	8.0	-12.36	0.0001868	3.5677	5.24621	0.0356	1.629	4.79E+01	5.490665
Y	1	4.1	-18.46	-0.0019089	2.9564	5.82268	0.04045	-1.8949	2.24E+01	6.101965
Y	2	0.9	6.38	-0.0019089	2.9564	5.82268	0.03495	-1.8949	7.73E+00	6.101965
4FOH	1	3.2	-19.26	-0.0023598	2.837	5.95240	0.04295	1.4147	2.10E+01	6.221365
4FOH	2	0.7	6.39	-0.0023598	2.837	5.95240	0.03895	1.4147	6.96E+00	6.221365
34OHF	2	0.8	6.78	-0.0015925	3.1654	5.59275	0.0304	-2.2406	8.99E+00	5.892965
34OHF	4	9.3	-13.72	-0.0015925	3.1654	5.59275	0.0422	-2.2406	1.82E+01	5.892965
34OHF	5	3.5	1.96	-0.0015925	3.1654	5.59275	0.0364	-2.2406	2.60E+00	5.892965
3NY	2	1.2	2.48	-0.0009019	3.3638	5.15409	0.0298	-2.5722	4.37E+00	5.694565
3NY	4	16.0	-4.43	-0.0009019	3.3638	5.15409	0.0413	-2.5722	7.81E+00	5.694565

3NY	5	7.0	-7.49	-0.0009019	3.3638	5.15409	0.0325	-2.5722	1.32E+01	5.694565
3MOH	1	2.7	-17.03	-0.0019808	2.9225	5.86602	0.0357	1.6578	2.03E+01	6.135865
3MOH	3	8.9	-28.42	-0.0019808	2.9225	5.86602	0.0543	1.6578	3.38E+01	6.135865
3MOH	4	0.8	7.55	-0.0019808	2.9225	5.86602	0.0373	1.6578	8.98E+00	6.135865
3MOH	5	3.7	-21.26	-0.0019808	2.9225	5.86602	0.0481	1.6578	2.53E+01	6.135865
3OHCOOH	1	0.6	-21.78	-0.0022875	2.5437	6.25172	0.0412	0.799	2.41E+01	6.514665
3OHCOOH	3	1.6	-26.40	-0.0022875	2.5437	6.25172	0.0481	0.799	2.92E+01	6.514665
3OHCOOH	4	0.5	7.31	-0.0022875	2.5437	6.25172	0.0371	0.799	8.09E+00	6.514665
3OHCOOH	5	1.0	-18.86	-0.0022875	2.5437	6.25172	0.0407	0.799	2.09E+01	6.514665
4MOH	1	9.1	-17.15	-0.0018317	3.0534	5.73094	0.0414	1.6578	2.12E+01	6.004965
4MOH	2	1.4	5.84	-0.0018317	3.0534	5.73094	0.03725	1.6578	7.23E+00	6.004965
4OMOH	1	10.3	-13.54	-0.0018315	3.3901	5.36599	0.03765	1.2439	1.67E+01	6.004965
4OMOH	2	3.5	2.60	-0.0018315	3.3901	5.36599	0.03715	1.2439	3.21E+00	6.004965
W	2	6.7	-8.73	0.0005291	3.4664	5.27323	0.0396	-1.5098	2.01E+01	5.591965
W	4	4.2	-15.51	0.0005291	3.4664	5.27323	0.0352	-1.5098	3.57E+01	5.591965
W	5	23.8	0.33	0.0005291	3.4664	5.27323	0.0291	-1.5098	7.52E-01	5.591965
W	6	2.8	-8.75	0.0005291	3.4664	5.27323	0.0336	-1.5098	2.01E+01	5.591965
W	7	22.7	-8.99	0.0005291	3.4664	5.27323	0.0336	-1.5098	2.07E+01	5.591965
3FY	2	2.5	6.97	-0.0018303	2.8019	5.97687	0.033	-1.7941	8.63E+00	6.256465
3FY	4	3.4	5.01	-0.0018303	2.8019	5.97687	0.0373	-1.7941	6.20E+00	6.256465
3FY	5	8.8	-17.24	-0.0018303	2.8019	5.97687	0.0392	-1.7941	2.13E+01	6.256465
4OHCOOH	1	1.3	-19.51	-0.0025813	2.4634	6.34922	0.0412	0.799	2.03E+01	6.594965
4OHCOOH	2	0.7	8.48	-0.0025813	2.4634	6.34922	0.0325	0.799	8.84E+00	6.594965
2M6F	3	105.5	-25.59	0.0003205	3.515	5.23862	0.0539	2.1437	7.57E+01	5.543365
2M6F	5	4.3	-14.64	0.0003205	3.515	5.23862	0.0341	2.1437	4.33E+01	5.543365
2M6F	4	17.4	5.18	0.0003205	3.515	5.23862	0.0268	2.1437	1.53E+01	5.543365
2M6F	7	6.9	-2.26	0.0003205	3.515	5.23862	0.0315	2.1437	6.69E+00	5.543365
P	1	14.3	-14.42	-0.0016864	3.3121	5.41963	0.0343	1.0176	1.86E+01	5.746265
P	2	2.1	3.29	-0.0016864	3.3121	5.41963	0.03375	1.0176	4.24E+00	5.746265
2MOH	2	1.5	7.96	-0.0018288	2.9248	5.86601	0.0348	1.6578	9.86E+00	6.133565
2MOH	3	11.9	-25.68	-0.0018288	2.9248	5.86601	0.0531	1.6578	3.18E+01	6.133565
2MOH	4	1.1	5.96	-0.0018288	2.9248	5.86601	0.0399	1.6578	7.37E+00	6.133565
2MOH	5	6.1	-17.22	-0.0018288	2.9248	5.86601	0.0405	1.6578	2.13E+01	6.133565

Table S3. Analysis of the electron transfer mechanism for molecules containing phenyl structure.

Molecule	Atom	Absolute SNE	$g_{Molecule} - g_{Dye}$ (a.u.) for PCET	$g_{Molecule} - g_{Dye}$ (a.u.) for ET	a_{iso} (MHz) for PCET	a_{iso} (MHz) for ET	Kaptein rules for PCET[a]	Kaptein rules for ET[a]	Experimental sign[a]	Mechanism
Y	1	4.1	0.0019089	0.0000609	-18.46	-9.29	-1	-1	-1	PCET
Y	2	0.9	0.0019089	0.0000609	6.38	-0.83	1	-1	1	
4FOH	1	3.2	0.0023598	0.0006293	-19.26	-10.80	-1	-1	-1	PCET
4FOH	2	0.7	0.0023598	0.0006293	6.39	-0.16	-1<br>$a_{iso}(1)*a_{iso}(2)$	1<br>$a_{iso}(1)*a_{iso}(2)$	A/E (-1)	
34OHF	2	1.2	0.0015925	0.0002780	6.78	3.85	1	1	-1	PCET
34OHF	4	9.3	0.0015925	0.0002780	-13.72	-12.49	-1	-1	-1	
34OHF	5	3.5	0.0015925	0.0002780	1.96	0.40	-1<br>$a_{iso}(4)*a_{iso}(5)$	-1<br>$a_{iso}(4)*a_{iso}(5)$	A/E (-1)	
3NY	2	1.2	0.0009019	-0.0000902	2.48	0.30	1	-1	1	PCET
3NY	4	16.0	0.0009019	-0.0000902	-4.43	-15.65	-1	1	-1	
3NY	5	7.0	0.0009019	-0.0000902	-7.49	3.61	-1	-1	-1	
3MOH	1	2.7	0.0019808	-0.0000424	-17.03	-4.53	-1	1	-1	PCET
3MOH	3	8.9	0.0019808	-0.0000424	-28.42	-29.09	-1	1	-1	
3MOH	4	0.8	0.0019808	-0.0000424	7.55	4.21	1	-1	1	
3MOH	5	3.7	0.0019808	-0.0000424	-21.26	-18.04	-1	1	-1	
3OHCOOH	1	0.6	0.0022875	0.0001105	-21.78	-14.70	-1	-1	-1	PCET
3OHCOOH	3	1.6	0.0022875	0.0001105	-26.40	-26.31	-1	-1	-1	
3OHCOOH	4	1.0	0.0022875	0.0001105	7.31	1.48	1	1	1	
3OHCOOH	5	0.5	0.0022875	0.0001105	-18.86	-10.66	-1	-1	1	
4MOH	1	9.1	0.0018317	-0.0000194	-17.15	-9.06	-1	1	-1	PCET
4MOH	2	1.4	0.0018317	-0.0000194	5.84	-1.50	-1<br>$a_{iso}(1)*a_{iso}(2)$	1<br>$a_{iso}(1)*a_{iso}(2)$	A/E (-1)	
4OMOH	1	10.3	0.0018315	0.0006214	-13.54	-4.65	-1	-1	-1	PCET
4OMOH	2	3.5	0.0018315	0.0006214	2.60	-6.26	-1<br>$a_{iso}(1)*a_{iso}(2)$	1<br>$a_{iso}(1)*a_{iso}(2)$	A/E (-1)	
2MOH	2	1.5	0.0018288	0.0000264	7.96	4.14	-1<br>$a_{iso}(2)*a_{iso}(3)$	-1<br>$a_{iso}(2)*a_{iso}(3)$	A/E(-1)	PCET
2MOH	3	11.9	0.0018288	0.0000264	-25.68	-24.75	-1	-1	-1	
2MOH	4	1.1	0.0018288	0.0000264	5.96	-2.23	1	-1	1	
2MOH	5	6.1	0.0018288	0.0000264	-17.22	-7.19	-1	-1	-1	
3FY	2	2.5	0.0018303	0.0002797	6.97	1.01	1	1	1	PCET
3FY	4	3.4	0.0018303	0.0002797	5.01	-3.96	-1<br>$a_{iso}(4)*a_{iso}(5)$	1<br>$a_{iso}(4)*a_{iso}(5)$	E/A(1)	
3FY	5	8.8	0.0018303	0.0002797	-17.24	-6.12	-1	1	-1	
4OHCOOH	1	1.3	0.0025813	0.0007630	-19.51	-13.08	-1	-1	-1	PCET
4OHCOOH	2	0.7	0.0025813	0.0007630	8.48	3.53	1	1	1	
P	1	14.3	0.0016864	0.0005386	-14.42	-5.39	-1	-1	-1	PCET
P	2	2.1	0.0016864	0.0005386	3.29	-4.69	-1<br>$a_{iso}(1)*a_{iso}(2)$	1<br>$a_{iso}(1)*a_{iso}(2)$	A/E(-1)	
4OH	2	5.0	0.0010279	-0.0002283	-5.09	-4.67	-1	1	-1	PCET
4OH	3	29.0	0.0010279	-0.0002283	-7.75	-17.95	-1	1	-1	
4OH	5	2.5	0.0010279	-0.0002283	-18.47	-6.94	-1	1	-1	
4OH	6	13.5	0.0010279	-0.0002283	4.35	-5.03	1	1	-1	
4OH	7	47.0	0.0010279	-0.0002283	-25.38	-17.67	-1	1	-1	
5OH	2	0.7	0.0016573	-0.0001784	1.12	5.55	1	-1	1	PCET
5OH	3	1.0	0.0016573	-0.0001784	-5.48	-21.25	-1	1	-1	
5OH	4	31.9	0.0016573	-0.0001784	-22.03	-20.82	-1	1	-1	
5OH	6	5.8	0.0016573	-0.0001784	-10.27	2.75	-1	-1	-1	
5OH	7	0.5	0.0016573	-0.0001784	1.57	-12.60	1	1	1	
6OH	2	30.5	0.0012988	-0.0002675	-16.74	-18.26	-1	1	-1	PCET
6OH	3	2.8	0.0012988	-0.0002675	-0.98	-14.22	-1	1	-1	
6OH	4	0.4	0.0012988	-0.0002675	1.66	-10.00	1	1	1	
6OH	5	4.2	0.0012988	-0.0002675	-8.59	2.81	-1	-1	-1	
6OH	7	29.0	0.0012988	-0.0002675	-19.12	-8.85	-1	1	-1	
7OH	2	0.9	0.0010661	-0.0002575	4.06	-0.10	1	1	1	PCET
7OH	3	2.3	0.0010661	-0.0002575	-6.93	-18.68	-1	1	-1	
7OH	4	49.2	0.0010661	-0.0002575	-27.96	-22.77	-1	1	-1	
7OH	5	4.6	0.0010661	-0.0002575	4.35	0.16	-1<br>$a_{iso}(4)*a_{iso}(5)$	-1<br>$a_{iso}(4)*a_{iso}(5)$	A/E (-1)	
7OH	6	21.9	0.0010661	-0.0002575	-20.10	-13.85	-1	1	-1	

[a] Calculated with eq. 2 for the absorptive and emissive signals and with eq. 3 for multiplets. For multiplets the vicinal protons (in brackets) were taken into accounts in terms of a_{iso}. J couplings for protons in aromatics systems are generally positive[58]. The entries marked in green indicate the calculations that match the experimental sign.

Table S4. Diffusion coefficients for the compound library, determined by DOSY NMR

Molecule	Diffusion coefficient (m^2/s)[a]	Molecule	Diffusion coefficient (m^2/s)[a]
2M5F	1.26E-04	Y	8.87E-05
I	1.30E-04	4FOH	1.27E-04
4F	1.30E-04	34OHF	8.62E-05
5F	1.29E-04	3NY	8.55E-05
6F	1.30E-04	3MOH	1.21E-04
7F	1.22E-04	3OHCOOH	1.04E-04
4M	1.19E-04	4MOH	1.19E-04
5M	1.17E-04	4OMOH	1.15E-04
6M	1.21E-04	2MOH	1.22E-04
7M	1.16E-04	W	2.50E-04
4OH	1.14E-04	3FY	9.42E-05
5OH	1.17E-04	4OHCOOH	9.35E-05
6OH	1.14E-04	2M6F	1.15E-04
7OH	1.14E-04	P	9.90E-05
4COOH	9.97E-05		
5COOH	9.75E-05		
6COOH	9.67E-05		
7COOH	2.79E-04		
4N	1.19E-04		
5N	1.21E-04		
6N	1.23E-04		
7N	1.19E-04		
4OM	1.14E-04		
5OM	1.19E-04		
6OM	1.17E-04		
7OM	1.15E-04		

[a] measured on samples containing 1 mM of compound in 100 mM phosphate buffer (pH=7.1). DOSY experiments were measured with 4 dummy scans, 16 scans and 1 s of recycle delay, with stimulated echo using bipolar gradient pulses for diffusion and excitation sculpting for water suppression.

Table S5. T_1 relaxation time constants for all molecules in the compound library, measured for series of samples containing 1 mM of compounds in 100 mM phosphate buffer (pH=7.1). T_1 were measured with 4 dummy scans, 8 scans and 20 s of recycle delay using an inversion recovery experiment with excitation sculpting.

Molecule	Atom	T_1 (s)	Molecule	Atom	T_1 (s)	Molecule	Atom	T_1 (s)	Molecule	Atom	T_1 (s)	Molecule	Atom	T_1 (s)
2M5F	3	5.404	5M	2	4.986	4COOH	2	4.171	4OM	2	4.846	4MOH	1	4.385
2M5F	4	4.465	5M	3	5.813	4COOH	3	4.508	4OM	3	5.212	4MOH	2	4.223
2M5F	6	4.644	5M	4	5.806	4COOH	5	4.043	4OM	5	3.516	4OMOH	1	4.018
2M5F	7	5.734	5M	6	4.723	4COOH	6	3.214	4OM	6	3.871	4OMOH	2	3.935
I	2	7.084	5M	7	4.986	4COOH	7	3.879	4OM	7	4.284	2MOH	2	4.74
I	3	7.31	6M	2	5.209	5COOH	2	4.069	5OM	7	4.513	2MOH	3	4.659
I	7	6.428	6M	3	6.26	5COOH	3	4.323	5OM	3	5.248	2MOH	4	4.584
I	5	6.201	6M	4	5.158	5COOH	4	3.457	5OM	4	4.135	2MOH	5	4.497
I	6	6.291	6M	5	4.78	5COOH	7	4.772	5OM	6	4.475	W	2	2.788
I	4	6.65	6M	7	5.746	5COOH	6	3.496	5OM	2	5.214	W	4	3.263
4F	2	6.31	7M	2	5.521	6COOH	2	4.074	6OM	2	5.05	W	6	2.278
4F	3	6.932	7M	3	6.212	6COOH	3	4.762	6OM	3	5.521	W	5	2.52
4F	5	6.344	7M	4	5.48	6COOH	4	3.476	6OM	4	4.578	W	7	2.287
4F	6	5.734	7M	5	4.771	6COOH	5	3.579	6OM	5	4.415	4OHCOOH	1	3.592
4F	7	5.933	4OH	2	4.43	6COOH	7	4.808	6OM	7	3.99	4OHCOOH	2	3.864
5F	2	6.167	4OH	3	3.678	7COOH	2	4.06	7OM	2	5.194	P	1	3.07
5F	3	6.48	4OH	5	4.709	7COOH	3	5.278	7OM	3	5.768	P	2	3.161
5F	7	6.552	4OH	6	3.594	7COOH	4	4.017	7OM	4	4.873			
5F	6	5.838	4OH	7	4.023	7COOH	5	3.312	7OM	5	4.209			
5F	4	5.678	5OH	2	4.895	7COOH	6	4.279	7OM	6	3.736			
6F	2	6.639	5OH	3	5.136	4N	2	5.24	Y	1	2.486			
6F	3	6.973	5OH	4	4.959	4N	3	5.043	Y	2	1.937			
6F	4	6.953	5OH	6	4.085	4N	6	4.73	4FOH	1	5.257			
6F	5	6.432	5OH	7	4.298	5N	2	5.159	4FOH	2	5.59			
6F	7	6.309	6OH	2	4.523	5N	3	5.324	34OHF	2	1.331			
7F	2	6.649	6OH	3	5.021	5N	4	4.541	34OHF	4	1.333			
7F	3	7.454	6OH	4	4.202	5N	6	3.862	34OHF	5	1.467			
7F	4	6.468	6OH	5	3.921	5N	7	4.463	3NY	2	1.698			
7F	5	6.925	6OH	7	4.496	6N	2	5.152	3NY	4	1.707			
7F	6	6.05	7OH	2	4.592	6N	3	5.679	3NY	5	1.814			
4M	2	5.847	7OH	3	5.225	6N	7	4.638	3MOH	1	4.574			
4M	3	5.692	7OH	4	4.393	6N	5	4.167	3MOH	3	4.509			
4M	5	5.113	7OH	5	3.767	6N	4	4.775	3MOH	4	4.328			
4M	6	4.985	7OH	6	3.851	7N	2	5.199	3MOH	5	4.577			
4M	7	5.711	2M6F	3	5.364	7N	3	5.786	3OHCOOH	1	3.974			
3FY	2	2.282	2M6F	5	4.844	7N	4	5.129	3OHCOOH	3	3.624			
3FY	4	2.041	2M6F	4	4.752	7N	5	4.409	3OHCOOH	4	3.195			
3FY	5	2.041	2M6F	7	5.211	7N	6	4.407	3OHCOOH	5	3.747			

Table S6. Mulliken and Löwdin spin densities and Hirshfeld charges of all protons in the compound library.

Molecule	Atom	Mulliken Spin Density	Löwdin Spin Density	Hirshfeld charges	Molecule	Atom	Mulliken Spin Density	Löwdin Spin Density	Hirshfeld charges	Molecule	Atom	Mulliken Spin Density	Löwdin Spin Density	Hirshfeld charges
2M5F	3	-0.026956	0.012319	0.1332261	7OH	4	-0.030423	0.012824	0.1233642	34OHF	2	0.007569	-0.003917	0.1449576
2M5F	4	-0.015632	0.006301	0.1402109	7OH	5	0.004070	-0.002312	0.1224309	34OHF	4	0.001439	-0.001092	-0.272626
2M5F	6	-0.017929	0.007337	0.1349154	7OH	6	-0.020307	0.007455	0.1181200	34OHF	5	-0.013201	0.004012	0.1284522
2M5F	7	0.002657	-0.001124	0.1457165	4COOH	2	-0.010727	0.004486	0.1644337	3NY	2	0.002988	-0.002374	-0.062202
I	2	-0.009083	0.004230	0.1561190	4COOH	3	-0.021436	0.011359	0.1138930	3NY	4	-0.006143	0.002192	0.0649880
I	3	-0.020321	0.010385	0.1350153	4COOH	5	0.007339	-0.002983	0.1119928	3NY	5	-0.007186	0.001555	0.1342135
I	7	-0.002052	0.000945	0.1366964	4COOH	6	-0.019349	0.007049	0.1339270	3MOH	1	-0.015780	0.005731	0.1348552
I	5	0.005724	-0.002685	0.1190199	4COOH	7	0.002086	-0.000791	0.1523613	3MOH	3	-0.032015	0.012970	0.1233837
I	6	-0.017628	0.006427	0.1223315	5COOH	2	-0.009210	0.003939	0.1648680	3MOH	4	0.008212	-0.003774	0.1331131
I	4	-0.018134	0.006708	0.1273896	5COOH	3	-0.021431	0.010920	0.1455856	3MOH	5	-0.020602	0.007559	0.1259950
4F	2	-0.007649	0.003459	0.1619650	5COOH	4	-0.016496	0.006202	0.1173624	3OHCOOH	1	-0.019256	0.007500	0.1283872
4F	3	-0.019496	0.009929	0.1382162	5COOH	7	-0.001356	0.000531	0.1511405	3OHCOOH	3	-0.017152	0.006136	0.1217406
4F	5	0.003578	-0.002130	0.1354849	5COOH	6	-0.017349	0.006684	0.1482657	3OHCOOH	4	0.007388	-0.003492	0.1463375
4F	6	-0.015844	0.005572	0.1327192	6COOH	2	-0.006398	0.002699	0.1674609	3OHCOOH	5	-0.026175	0.011445	0.1429312
4F	7	-0.005168	0.002315	0.1414906	6COOH	3	-0.022437	0.011498	0.1448500	4MOH	1	-0.016582	0.005734	0.1337889
5F	2	-0.007168	0.003329	0.1598166	6COOH	4	-0.018703	0.006709	0.1417072	4MOH	2	0.006091	-0.003163	0.1297682
5F	3	-0.022920	0.011614	0.1386488	6COOH	5	0.004767	-0.001985	0.1451848	4OMOH	1	-0.016978	0.005982	0.1383673
5F	7	-0.001722	0.000607	0.1481526	6COOH	7	-0.001586	0.000260	0.1260408	4OMOH	2	0.002215	-0.001742	0.1327890
5F	6	-0.013953	0.005758	0.1374490	7COOH	2	-0.011421	0.005173	0.1620995	W	2	-0.014091	0.004588	0.2053573
5F	4	-0.017014	0.006915	0.1428178	7COOH	3	-0.021554	0.010918	0.1430029	W	4	-0.015396	0.005927	0.1299035
6F	2	-0.012833	0.005855	0.1580460	7COOH	4	-0.014852	0.005270	0.1434944	W	5	0.004609	-0.002208	0.1187472
6F	3	-0.017057	0.008921	0.1387863	7COOH	5	0.005276	-0.002416	0.1313122	W	6	-0.015161	0.005365	0.1224910
6F	4	-0.015683	0.005437	0.1392071	7COOH	6	-0.012833	0.005309	0.1181888	W	7	-0.001395	0.000592	0.1368224
6F	5	0.005326	-0.002802	0.1348902	4N	2	-0.006065	0.002587	0.1509638	3FY	2	0.007066	-0.003564	0.1516526
6F	7	-0.002690	0.000622	0.1520192	4N	3	-0.007699	0.004212	0.1344646	3FY	4	0.004668	-0.002573	0.1380107
7F	2	-0.005545	0.002676	0.1621253	4N	6	-0.023004	0.009151	0.1176655	3FY	5	-0.016288	0.005405	0.1408728
7F	3	-0.019954	0.009909	0.1415081	5N	2	0.001217	-0.000879	0.1525676	4OHCOOH	1	-0.017989	0.006491	0.1395522
7F	4	-0.020548	0.007922	0.1324536	5N	3	-0.005876	0.001785	0.1297594	4OHCOOH	2	0.009087	-0.003967	0.1346898
7F	5	0.004137	-0.002164	0.1294305	5N	4	-0.016146	0.005460	0.1227366	2M6F	3	-0.026956	0.012319	0.1335691
7F	6	-0.015347	0.005727	0.1390070	5N	6	-0.006978	0.001138	0.3766838	2M6F	5	-0.015632	0.006301	0.1360443
4M	2	-0.009333	0.004092	0.1543201	5N	7	-0.003591	0.000314	0.3781249	2M6F	4	-0.017929	0.007337	0.1327300
4M	3	-0.019233	0.009628	0.1326635	6N	2	-0.017258	0.007719	0.1487966	2M6F	7	0.002657	-0.001124	0.1501131
4M	5	0.004623	-0.002237	0.1124429	6N	3	-0.002652	0.001878	0.1309565	P	1	-0.013661	0.004703	0.1403306
4M	6	-0.016756	0.005816	0.1199606	6N	7	-0.013754	0.004242	0.1322015	P	2	0.002209	-0.002149	0.1185538
4M	7	-0.004864	0.002177	0.1335876	6N	5	-0.004229	0.000401	0.1207133	2MOH	2	0.008895	-0.004038	0.1304347
5M	2	-0.008108	0.003487	0.1540727	6N	4	-0.003944	0.000689	0.1281769	2MOH	3	-0.029437	0.011659	0.1262903
5M	3	-0.021308	0.010712	0.1327368	7N	2	0.004805	-0.002280	0.1533340	2MOH	4	0.006245	-0.002978	0.1327574
5M	4	-0.020211	0.007462	0.1202020	7N	3	-0.009301	0.004016	0.1317892	2MOH	5	-0.016762	0.005639	0.1250376
5M	6	-0.014933	0.005580	0.1185433	7N	4	-0.027677	0.011383	0.1118818					
5M	7	-0.003979	0.001553	0.1353195	7N	5	0.002697	-0.001464	0.1146691					
6M	2	-0.011792	0.005233	0.1531961	7N	6	-0.019193	0.005720	0.1149364					

6M	3	-0.016365	0.008599	0.1331094	4OM	2	-0.008086	0.003609	0.1548634
6M	4	-0.017903	0.006092	0.1259784	4OM	3	-0.014133	0.006838	0.1274168
6M	5	0.006332	-0.002966	0.1153706	4OM	5	-0.002140	0.000352	0.1207017
6M	7	-0.003976	0.001246	0.1295410	4OM	6	-0.010528	0.003497	0.1234326
7M	2	-0.006509	0.002866	0.1339738	4OM	7	-0.013011	0.005432	0.1317283
7M	3	-0.019317	0.009564	0.1244056	5OM	7	-0.013501	0.003901	0.1395102
7M	4	-0.021178	0.008036	0.1546458	5OM	3	-0.010756	0.004098	0.1327925
4OH	2	-0.007240	0.003383	0.1576232	5OM	4	-0.013923	0.005263	0.1195561
4OH	3	-0.004307	0.002127	0.1421763	5OM	6	0.001499	-0.001571	0.1253555
4OH	5	-0.018889	0.006783	0.1160324	5OM	2	0.004751	-0.002514	0.1556993
4OH	6	0.003498	-0.002196	0.1253081	6OM	2	-0.015028	0.006617	0.1528900
4OH	7	-0.028149	0.011654	0.1320655	6OM	3	-0.009627	0.005330	0.1336691
5OH	2	0.001385	-0.000923	0.1553197	6OM	4	-0.012805	0.003925	0.1306371
5OH	3	-0.005828	0.001882	0.1332403	6OM	5	0.002852	-0.001828	0.1228236
5OH	4	-0.020544	0.008129	0.1364943	6OM	7	-0.008291	0.002589	0.1292771
5OH	6	-0.009645	0.002904	0.1203204	7OM	2	0.003136	-0.001488	0.1560419
5OH	7	-0.000082	-0.001142	0.141548	7OM	3	-0.015025	0.006816	0.1351051
6OH	2	-0.017140	0.007892	0.1523576	7OM	4	-0.024659	0.010137	0.1224246
6OH	3	0.000444	0.000197	0.1341585	7OM	5	-0.000377	-0.000491	0.1198056
6OH	4	0.000067	-0.000980	0.1325577	7OM	6	-0.012628	0.005213	0.1241534
6OH	5	-0.007954	0.002316	0.117509	Y	1	-0.017180	0.005860	0.1351182
6OH	7	-0.018529	0.007113	0.1455683	Y	2	0.006545	-0.003290	0.1358903
7OH	2	0.004695	-0.002364	0.1585515	4FOH	1	0.005079	-0.003224	0.1465205
7OH	3	-0.006681	0.002751	0.1375303	4FOH	2	-0.017925	0.006332	0.1519920

Table S7. Significance of the individual molecular features for semi-quantitative prediction of the photo-CIDNP SNE, determined with ANOVA.

Feature	F-statistic	p-value	Interpretation
f-	41.529264	2.38E-15	Significant
Q	29.330754	1.24E-11	Significant
a_{iso}	28.864757	1.75E-11	Significant
Δg	3.193422	4.36E-02	Significant
logP	2.990091	5.30E-02	Not Significant
IP	0.146794	8.64E-01	Not Significant
**LUMO-HOMO**	0.126173	8.82E-01	Not Significant
N	0.125323	8.82E-01	Not Significant

Table S8. Significance of the individual molecular features for semi-quantitative prediction of the photo-CIDNP SNE, determined with Tukey's test.

Feature	Category	Mean Difference	p-value	Interpretation
a_{iso}	90 ≥ SNE > 40 vs SNE > 90	0.039	0.9999	No significant difference between 90 ≥ SNE > 40 and SNE > 90.
	90 ≥ SNE > 40 vs SNE ≤ 40	15.136	0.0000	Significant difference between 90 ≥ SNE > 40 and SNE ≤ 40.
	SNE > 90 vs SNE ≤ 40	15.097	0.0000	Significant difference between SNE > 90 and SNE ≤ 40.
Δg	90 ≥ SNE > 40 vs SNE > 90	0.0003	0.1571	No significant difference between 90 ≥ SNE > 40 and SNE > 90.
	90 ≥ SNE > 40 vs SNE ≤ 40	-0.0000	0.9158	No significant difference between 90 ≥ SNE > 40 and SNE ≤ 40.
	SNE > 90 vs SNE ≤ 40	-0.0003	0.0286	Significant difference between SNE > 90 and SNE ≤ 40.
N	90 ≥ SNE > 40 vs SNE > 90	0.079	0.8768	No significant difference between 90 ≥ SNE > 40 and SNE > 90.
	90 ≥ SNE > 40 vs SNE ≤ 40	0.0307	0.9513	No significant difference between 90 ≥ SNE > 40 and SNE ≤ 40.
	SNE > 90 vs SNE ≤ 40	-0.0483	0.9285	No significant difference between SNE > 90 and SNE ≤ 40.
IP	90 ≥ SNE > 40 vs SNE > 90	-0.0886	0.8630	No significant difference between 90 ≥ SNE > 40 and SNE > 90.
	90 ≥ SNE > 40 vs SNE ≤ 40	-0.0610	0.8395	No significant difference between 90 ≥ SNE > 40 and SNE ≤ 40.
	SNE > 90 vs SNE ≤ 40	0.0276	0.9786	No significant difference between SNE > 90 and SNE ≤ 40.
f-	90 ≥ SNE > 40 vs SNE > 90	0.0037	0.3983	No significant difference between 90 ≥ SNE > 40 and SNE > 90.
	90 ≥ SNE > 40 vs SNE ≤ 40	-0.0120	0.0000	Significant difference between 90 ≥ SNE > 40 and SNE ≤ 40.
	SNE > 90 vs SNE ≤ 40	-0.0157	0.0000	Significant difference between SNE > 90 and SNE ≤ 40.
$logP$	90 ≥ SNE > 40 vs SNE > 90	0.5015	0.5954	No significant difference between 90 ≥ SNE > 40 and SNE > 90.
	90 ≥ SNE > 40 vs SNE ≤ 40	-0.4160	0.4101	No significant difference between 90 ≥ SNE > 40 and SNE ≤ 40.
	SNE > 90 vs SNE ≤ 40	-0.9175	0.0766	No significant difference between SNE > 90 and SNE ≤ 40.
**LUMO-HOMO**	90 ≥ SNE > 40 vs SNE > 90	-0.0790	0.8802	No significant difference between 90 ≥ SNE > 40 and SNE > 90.
	90 ≥ SNE > 40 vs SNE ≤ 40	-0.0281	0.9603	No significant difference between 90 ≥ SNE > 40 and SNE ≤ 40.
	SNE > 90 vs SNE ≤ 40	0.0509	0.9230	No significant difference between SNE > 90 and SNE ≤ 40.
Q	90 ≥ SNE > 40 vs SNE > 90	-26.4355	0.2228	No significant difference between 90 ≥ SNE > 40 and SNE > 90.
	90 ≥ SNE > 40 vs SNE ≤ 40	-47.3642	0.0000	Significant difference between 90 ≥ SNE > 40 and SNE ≤ 40.
	SNE > 90 vs SNE ≤ 40	-20.9288	0.2410	No significant difference between SNE > 90 and SNE ≤ 40.

Supplementary Figures

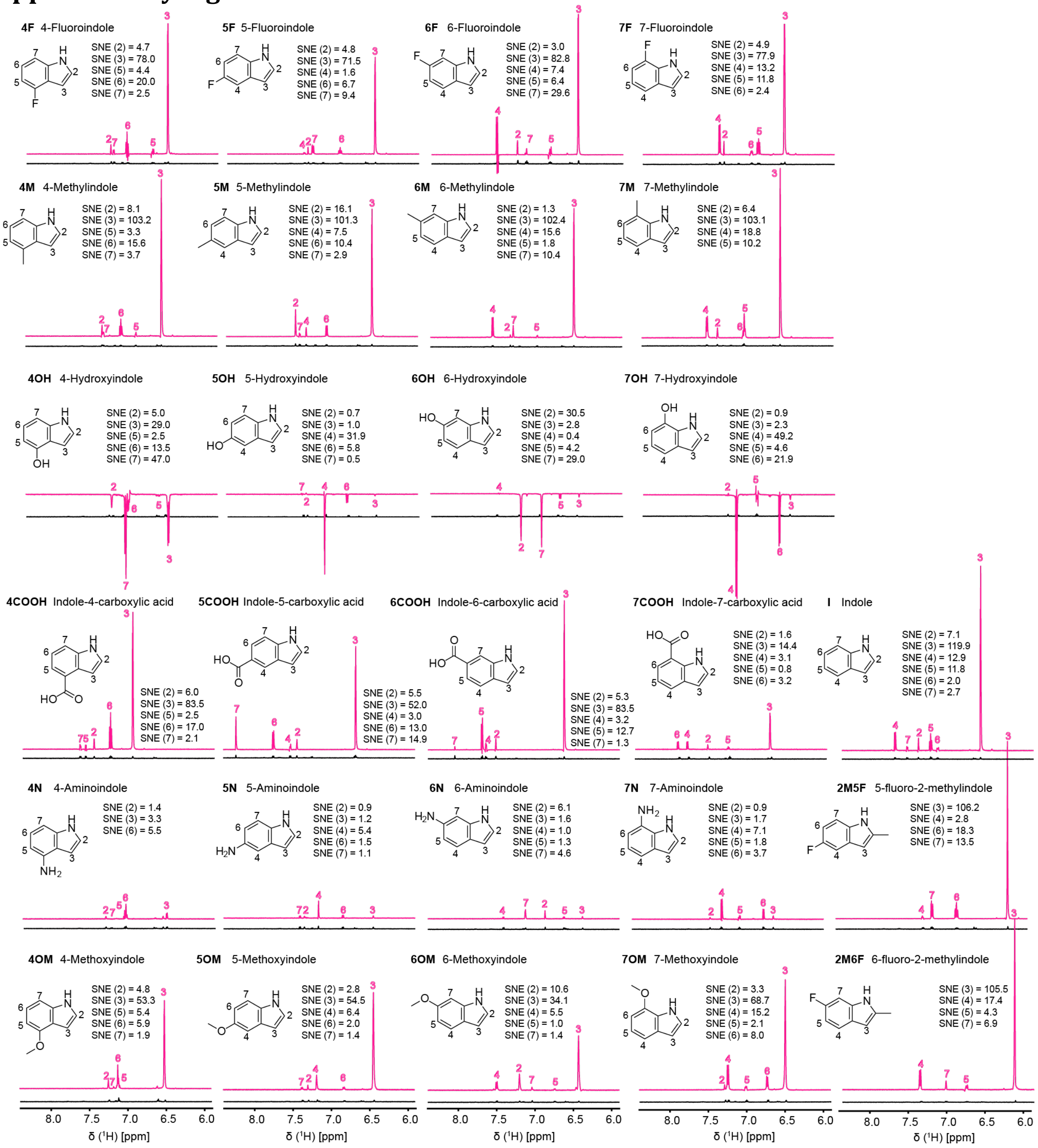

Figure S1. Laser irradiated (pink) and nonirradiated (black) 1D ^{1}H NMR spectra of indole derivatives with letter codes, structures with assigned protons, and corresponding SNEs. Laser irradiated spectra were recorded with 1 s of laser irradiation, 4 dummy scans, 16 scans and 3 s of recycle delay. The absorptive-emissive peaks originate from the multiplet effect.

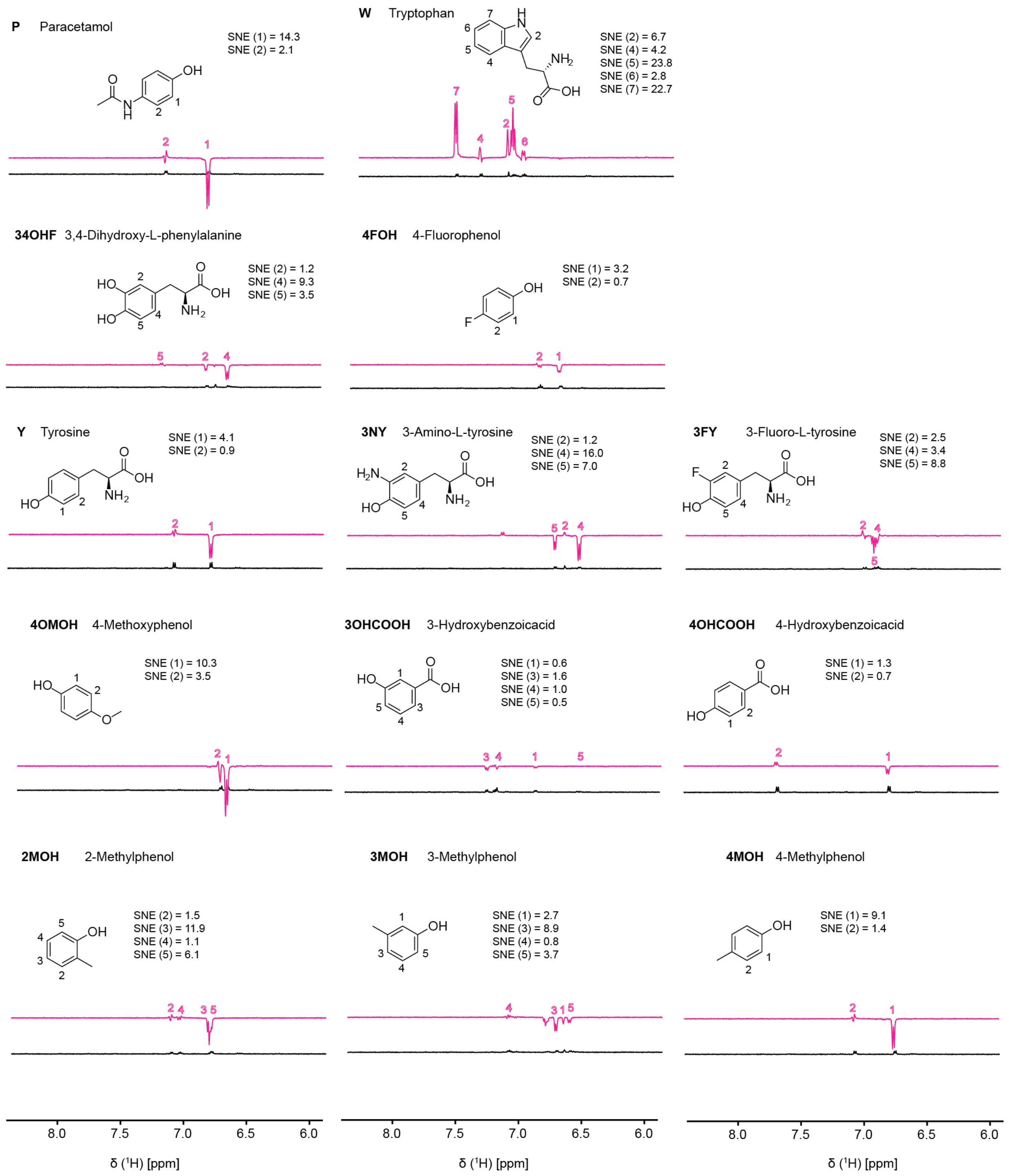

Figure S2. Laser irradiated (pink) and nonirradiated (black) 1D ^{1}H NMR spectra of amino acids and phenol derivatives with letter codes, structures with assigned protons, and corresponding SNEs. Laser irradiated spectra were recorded with 1 s of laser irradiation, 4 dummy scans, 16 scans, and 3 s of recycle delay. The absorptive-emissive peaks originate from the multiplet effect.

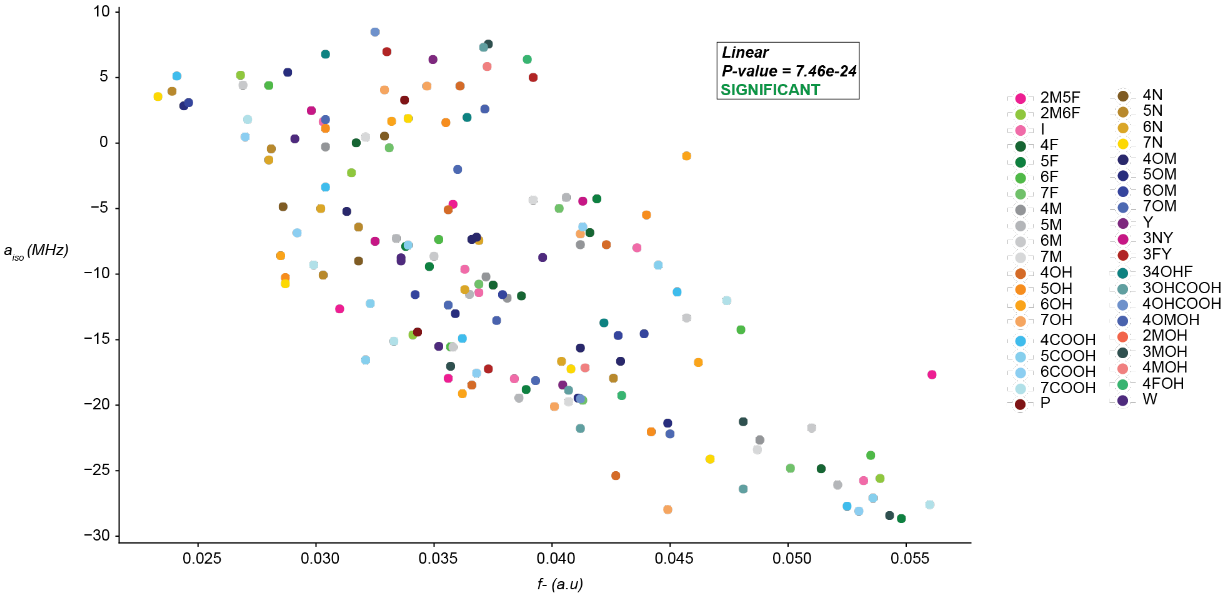

Figure S3. Correlation between isotropic hyperfine interactions, a_{iso}, and nucleophilic Fukui indices, *f-,* for individual sites in the compound library.

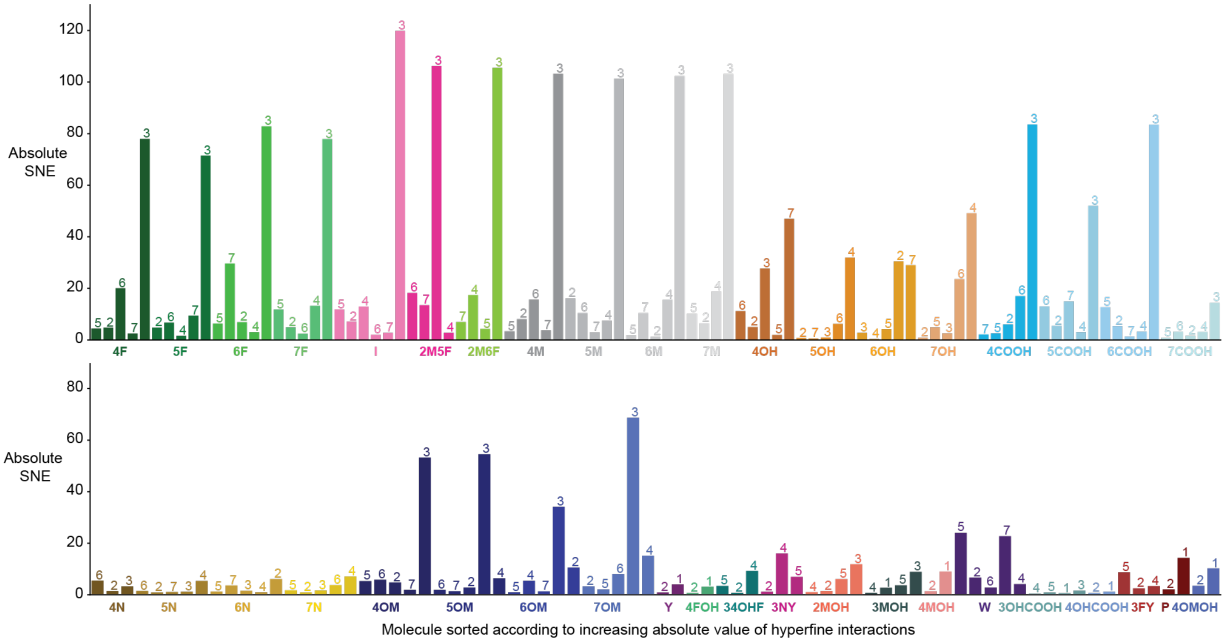

Figure S4. Hyperfine interactions as indicator of sites with high photo-CIDNP enhancement. Experimental photo-CIDNP effect for our compound library, given as absolute SNE. The abbreviations correspond to the individual molecules (Table S1) and numbers above the bars to the specific protons (Figure S1-S2). For each molecule, the sites are sorted by increasing hyperfine interactions. For 31 out of 40 tested molecules, the highest electron density corresponds to the highest enhancement within the molecule.

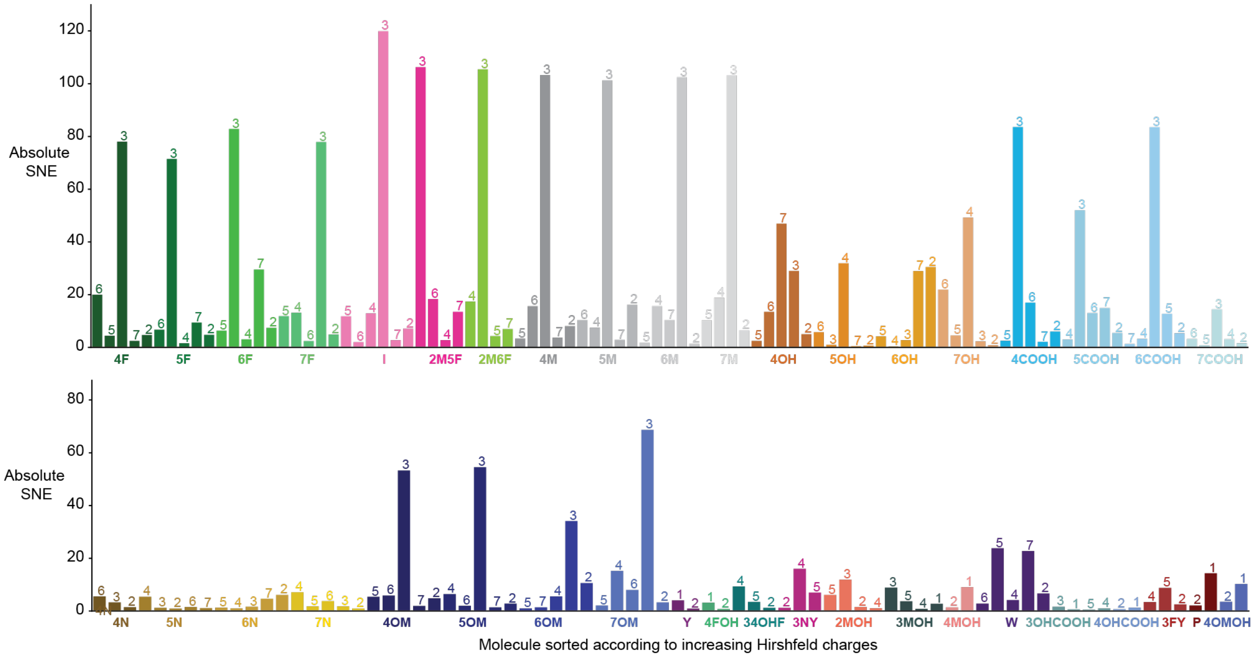

Figure S5. Hirshfeld charges as indicators of sites with high photo-CIDNP enhancement. Experimental photo-CIDNP effect for our compound library, given as absolute SNE. The abbreviations correspond to the individual molecules (Table S1) and numbers above the bars to the specific protons (Figure S1-S2). For each molecule, the sites are sorted by increasing Hirshfeld charges. Only for 7 out of 40 tested molecules, the highest electron density corresponds to the highest enhancement within the molecule.

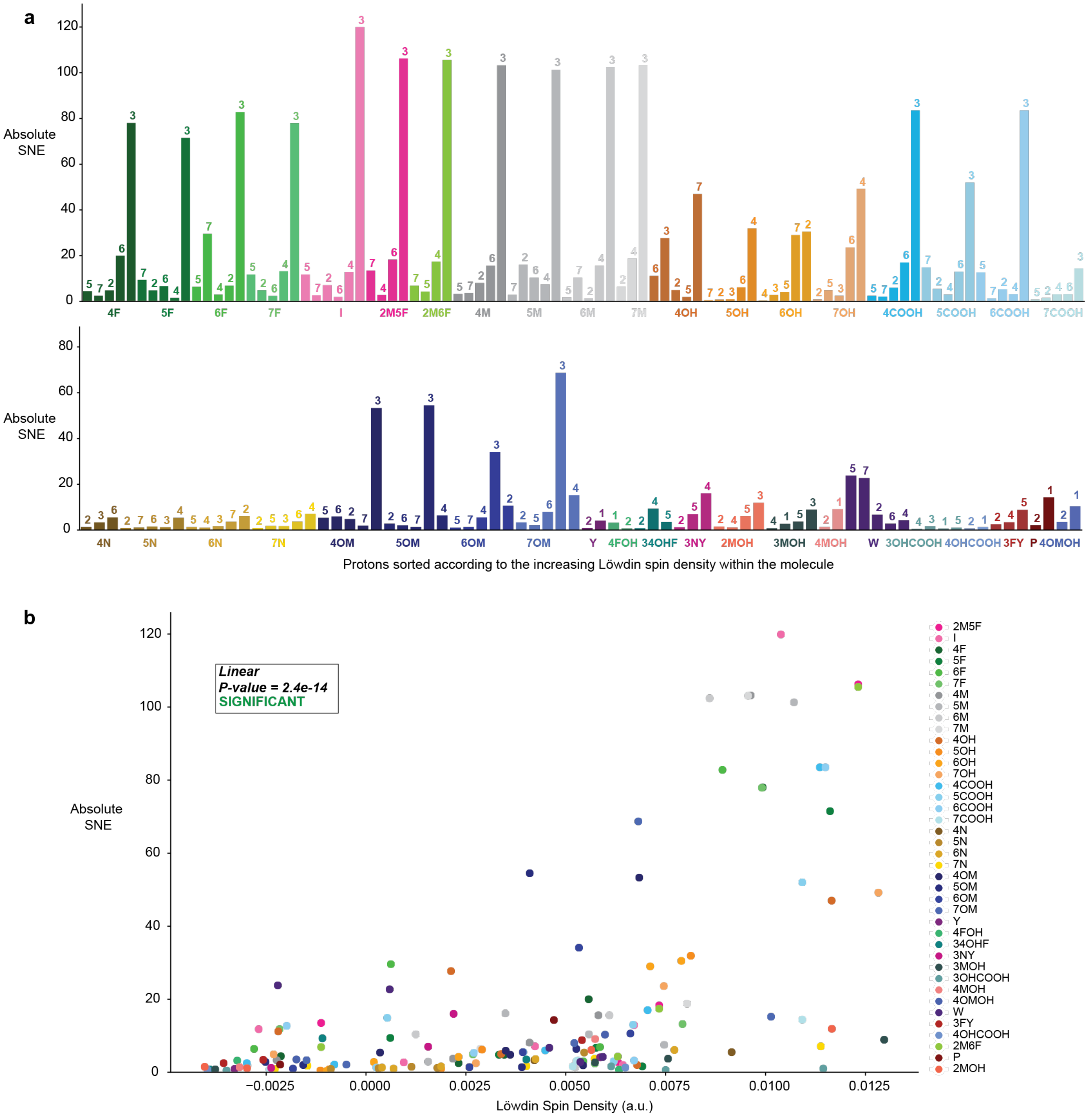

Figure S6. Löwdin spin densities as indicators of sites with high photo-CIDNP enhancement. (a) Experimental photo-CIDNP effect for our compound library, given as absolute SNE. The abbreviations correspond to the individual molecules (Table S1) and numbers above the bars to the specific protons (Figure S1-S2). For each molecule, the sites are sorted by increasing Löwdin spin densities. (b) Linear correlation between Löwdin spin densities with the photo-CIDNP effect. The Löwdin spin density is thus a worse indicator for photo-CIDNP than the nucleophilic Fukui index.

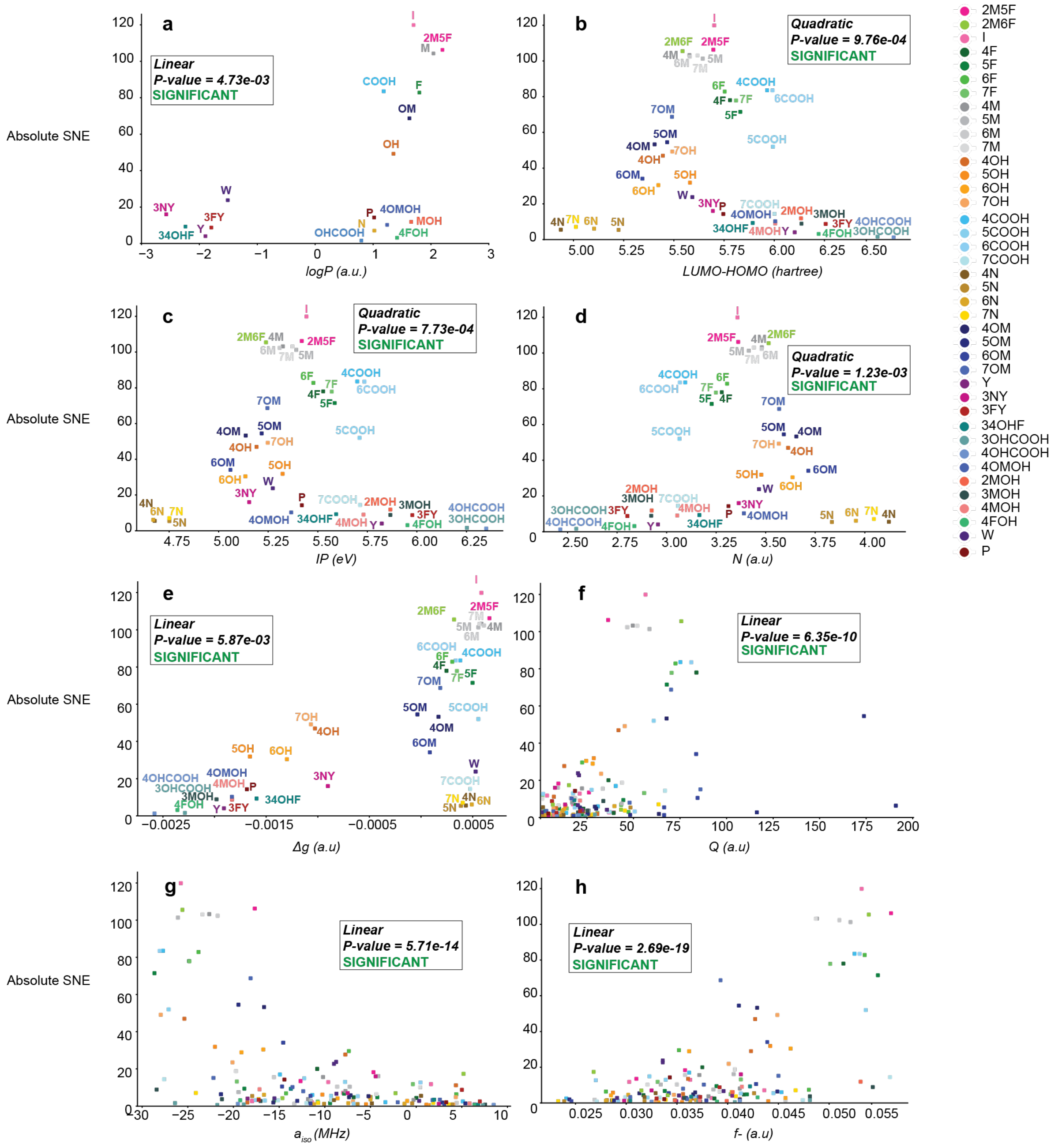

Figure S7. Linear and quadratic correlation of molecular features with the photo-CIDNP effect. The SNEs are presented the organizational level of the molecular feature: The highest SNE for derivative family for *logP* (a); the highest SNE of the individual molecule for the LUMO-HOMO energy gap (b), adiabatic ionization potential (c), the nucleophilicity index (d), *Δg* factor (e); the SNEs for individual protons with for the geminate polarization (d), isotropic hyperfine interactions (f), nucleophilic Fukui indices (g). The quadratic relationship was chosen for the electron-transfer-related properties in accordance with Marcus theory[36].

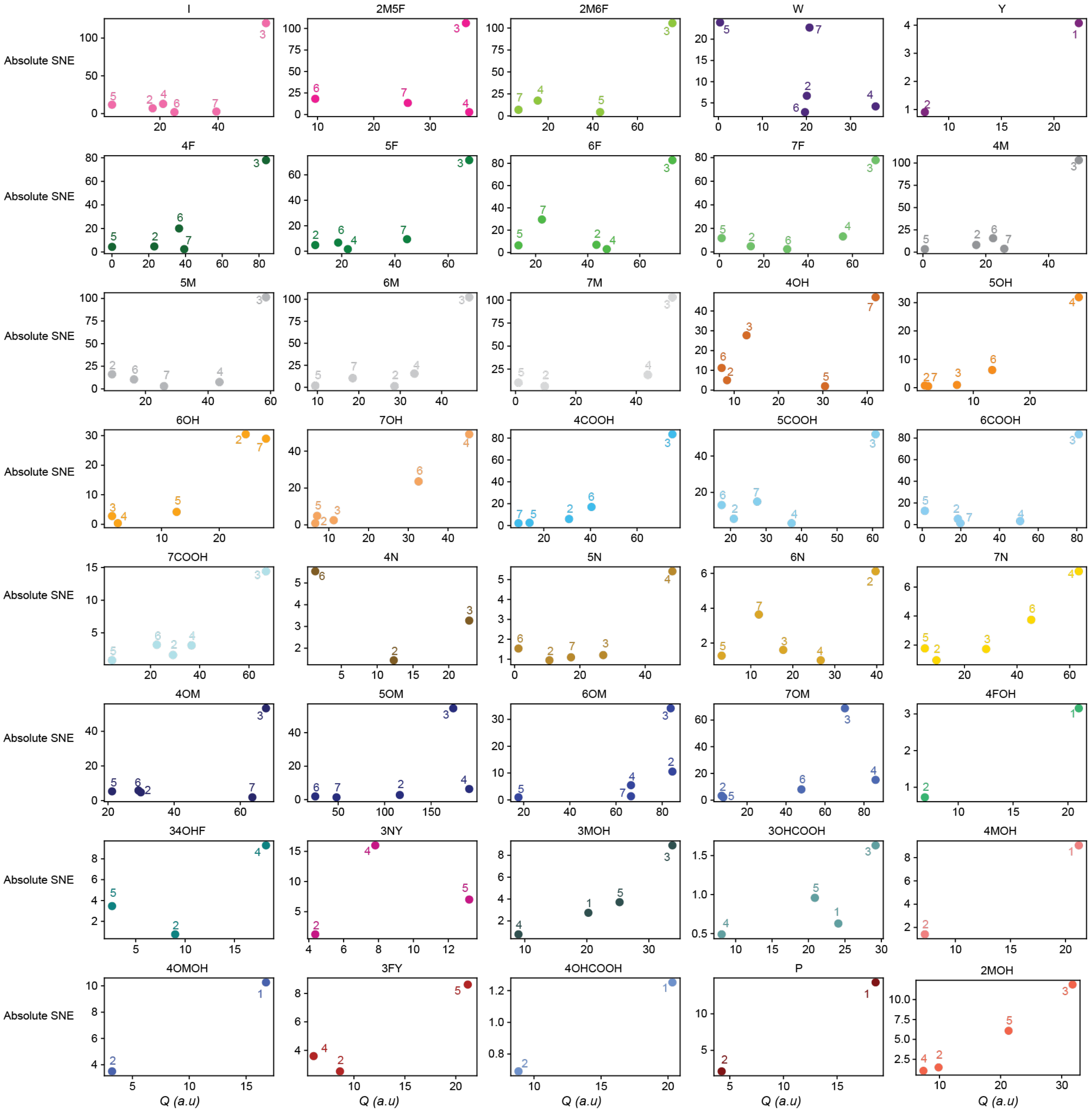

Figure S8. Zoom on the relationships between geminate polarization, *Q,* and photo-CIDNP enhancement, absolute SNE for individual molecules.

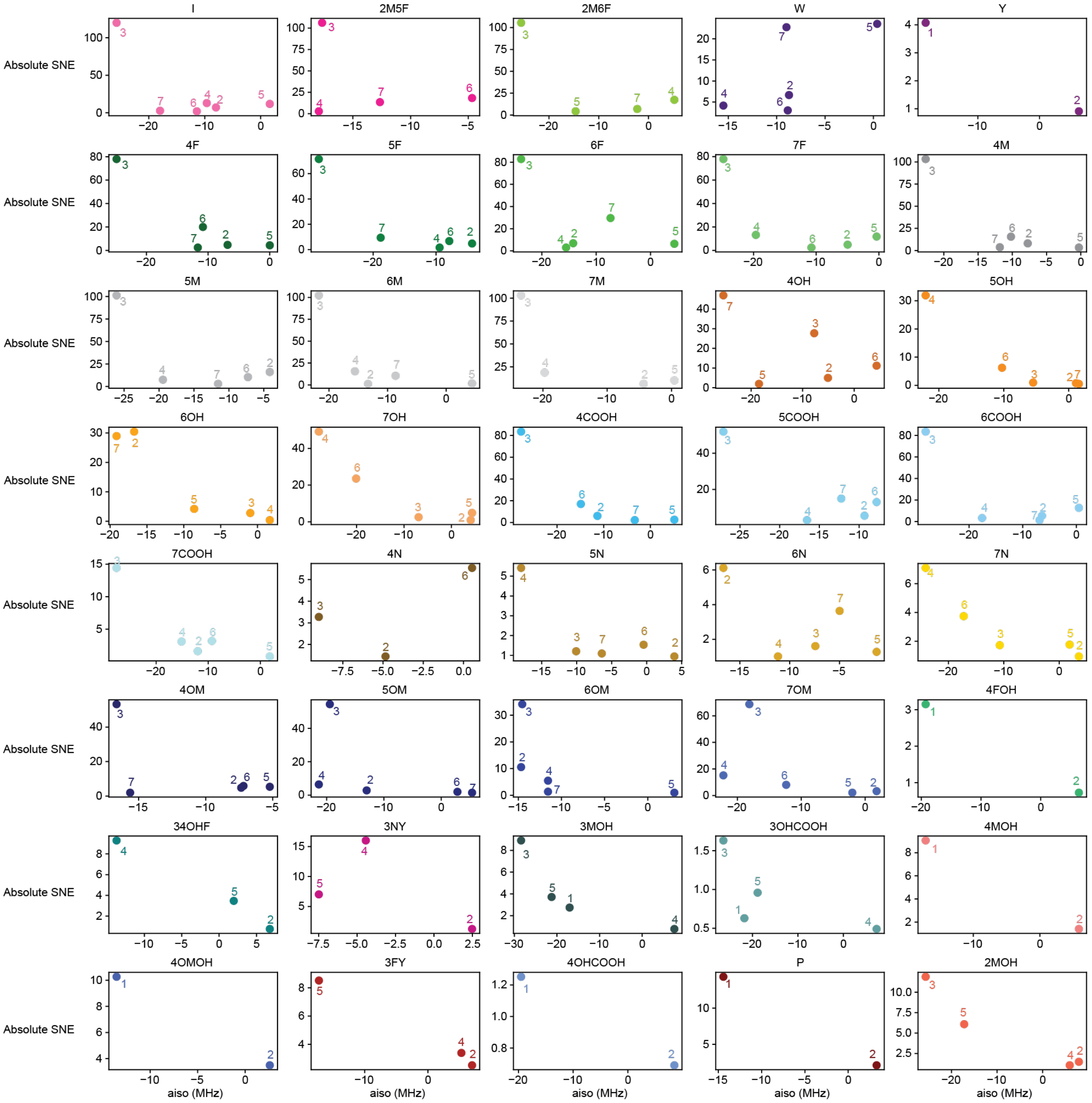

Figure S9. Zoom on the relationships between hyperfine interactions, a_{iso}, and photo-CIDNP enhancement, absolute SNE for individual molecules.

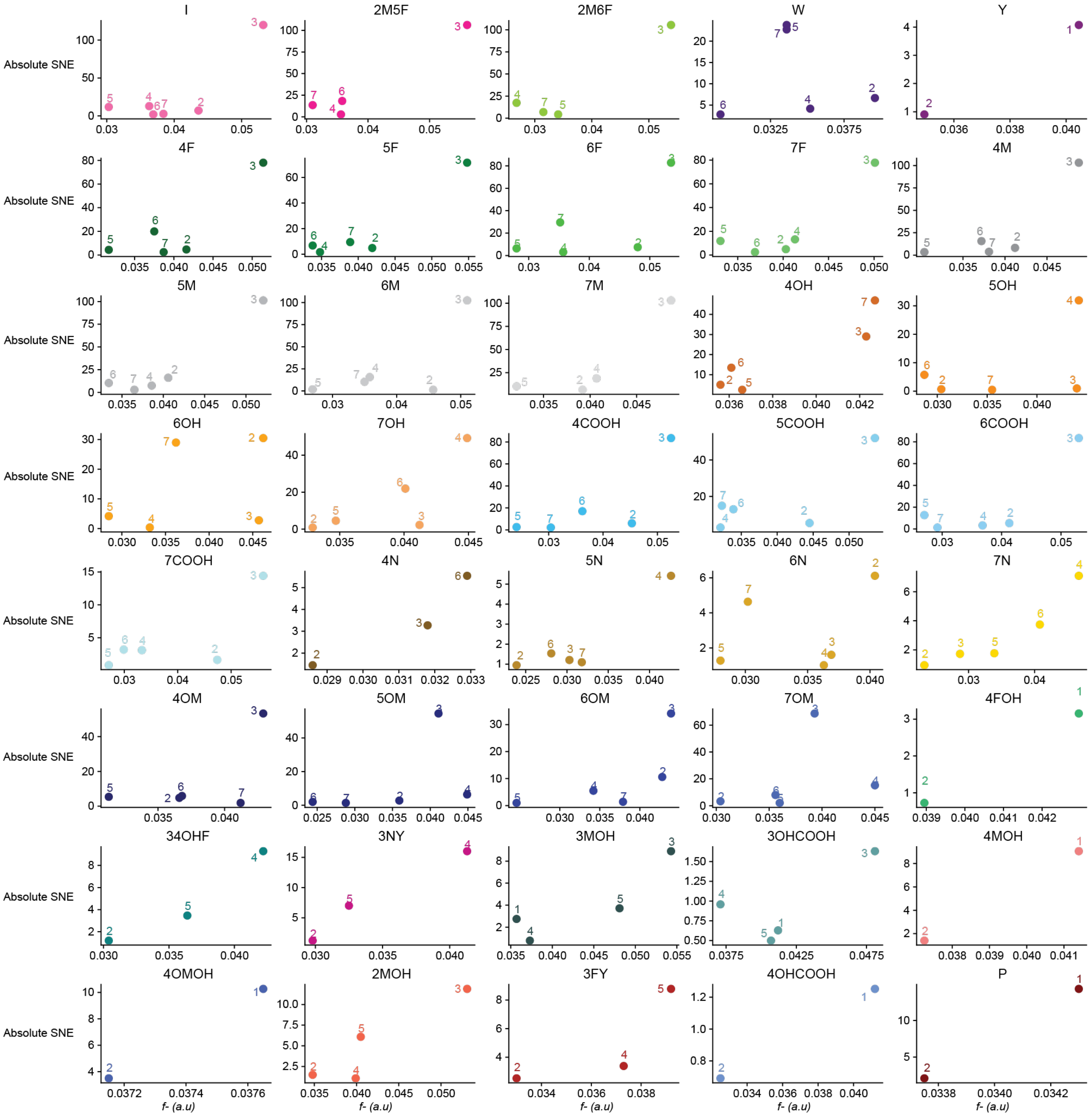

Figure S10. Zoom on the relationships between nucleophilic Fukui index, *f-*, and photo-CIDNP enhancement, absolute SNE for individual molecules.

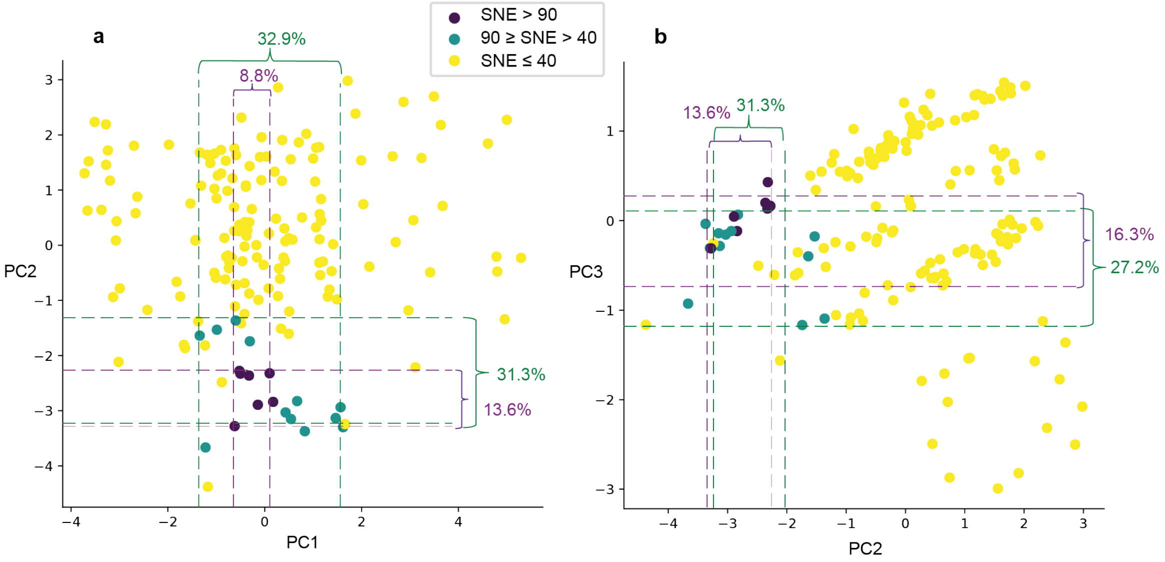

Figure S11. Grouping of protons based on Principal Component Analysis (PCA). (a) PC1 vs. PC2 (b) PC2 vs. PC3. Dashed lines represent the range of each category within the respective principal components. The percentage of the total range for each category in PC1, PC2, and PC3 is displayed next to the ranges.

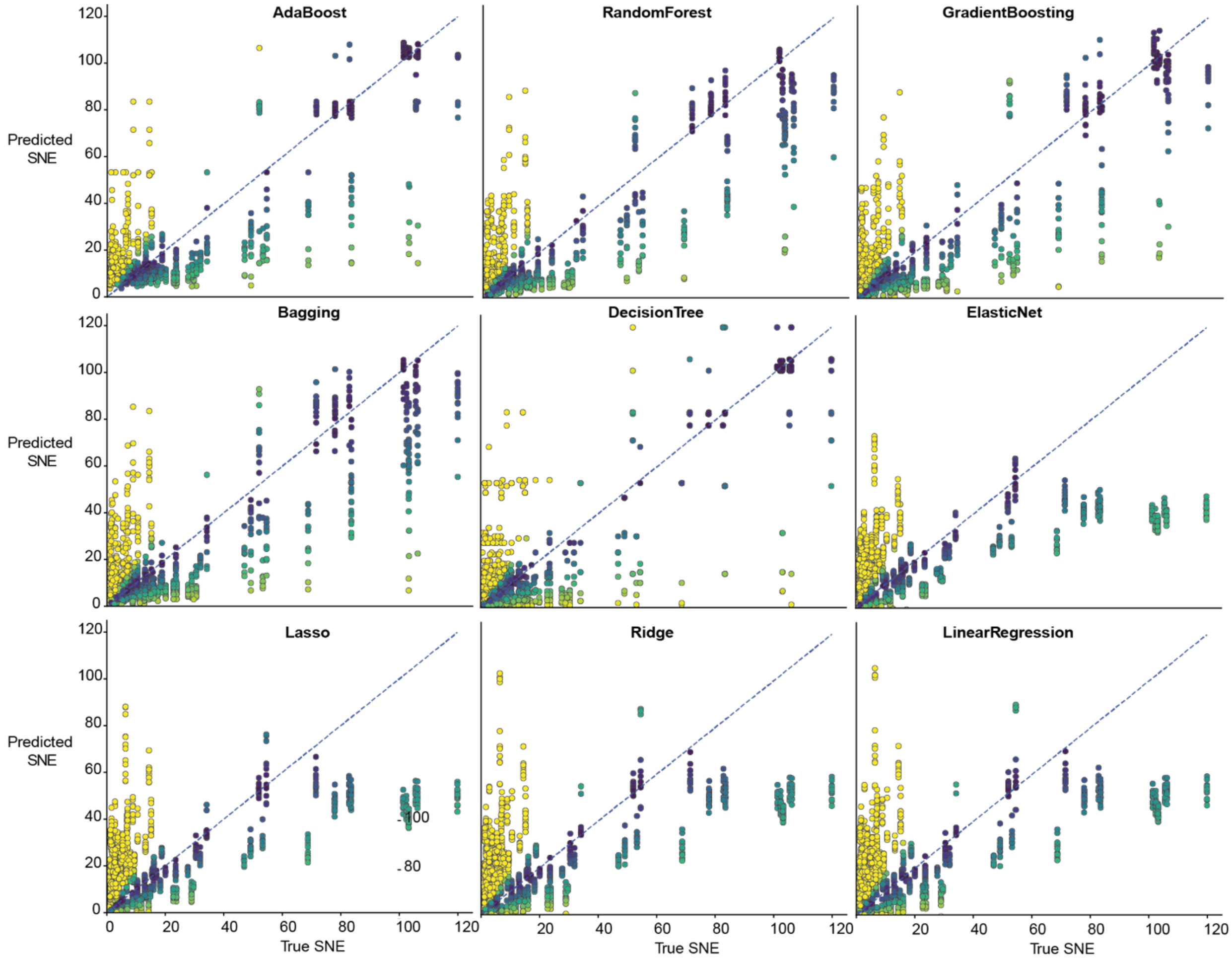

Figure S12. Correlation between predicted and experimentally determined SNE across 100 runs for tested quantitative machine learning models with percentage errors color scale.

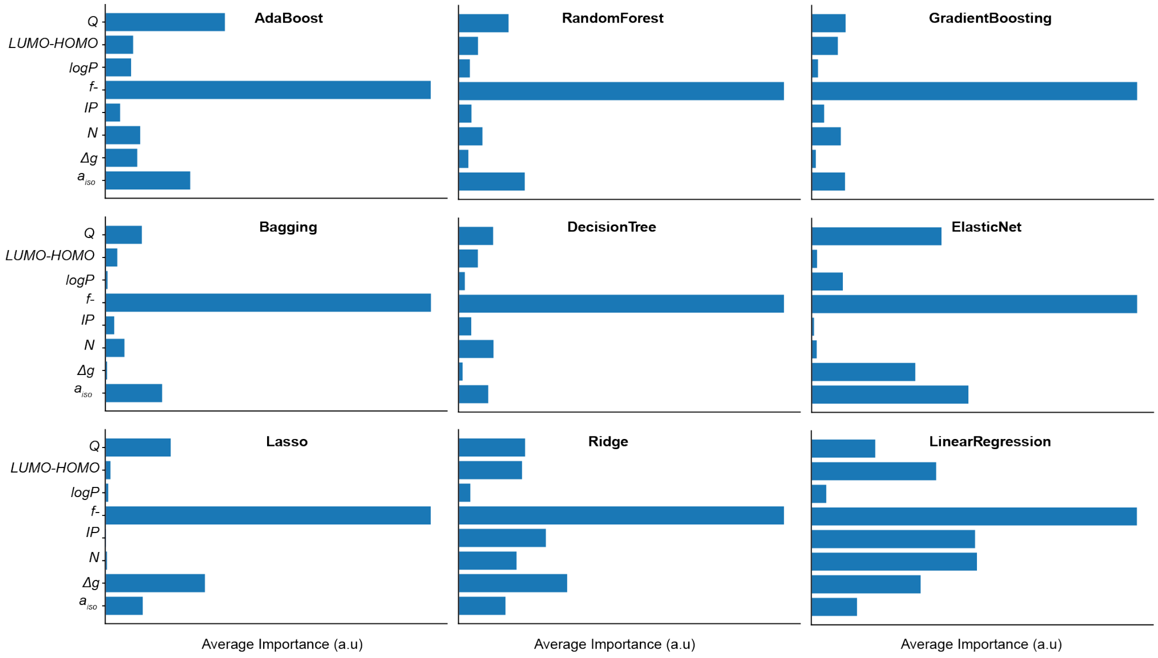

Figure S13. Averaged feature importance across 100 runs for tested quantitative machine learning models.

References

(41) Lee, J. H.; Cavagnero, S. A Novel Tri-Enzyme System in Combination with Laser-Driven NMR Enables Efficient Nuclear Polarization of Biomolecules in Solution. *J. Phys. Chem. B* **2013**, *117*, 6069–6081.

(42) Adams, R. W.; Holroyd, C. M.; Aguilar, J. A.; Nilsson, M.; Morris, G. A. “Perfecting” WATERGATE: Clean Proton NMR Spectra from Aqueous Solution. *Chem. Commun*. **2013**, *49* (4), 358–360.

(43) Bursch, M.; Mewes, J.-M.; Hansen, A.; Grimme, S. Best-Practice DFT Protocols for Basic Molecular Computational Chemistry. *Angew. Chem. Int. Ed.* **2022**, *61*, e202205735.

(44) Nottoli, M.; Herbst, M. F.; Mikhalev, A.; Jha, A.; Lipparini, F.; Stamm, B. ddX: Polarizable continuum solvation from small molecules to proteins. *WIREs Comput. Mol. Sci.* **2024**, *14*, e1726.

(45) Neese, F. Software update: The ORCA program system—Version 5.0. *WIREs Comput. Mol. Sci.* **2022**, *12*, e1606.

(46) Lu, T.; Chen, F. Multiwfn: A multifunctional wavefunction analyzer. *J. Comput. Chem.* **2012**, *33*, 580–592.

(47) Lu, T.; A Comprehensive Electron Wavefunction Analysis Toolbox for Chemists, Multiwfn. *J. Chem. Phys*. **2024**, *161*(8), 082503.

(48) Jakobsen, P.; Jensen, F. Probing basis set requirements for calculating hyperfine coupling constants. *J. Chem. Phys.* **2019**, *151*, 174107.

(49) Sander, T.; Freyss, J.; Von Korff, M.; Rufener, C. DataWarrior: An Open-Source Program For Chemistry Aware Data Visualization And Analysis. *J. Chem. Inf. Model.* **2015**, *55* (2), 460–473.

(50) Pedregosa, F.; Varoquaux, G.; Gramfort, A.; Michel, V.; Thirion, B.; Grisel, O.; Blondel, M.; Müller, A.; Nothman, J.; Louppe, G.; Prettenhofer, P.; Weiss, R.; Dubourg, V.; Vanderplas, J.; Passos, A.; Cournapeau, D.; Brucher, M.; Perrot, M.; Duchesnay, É. Scikit-learn: Machine Learning in Python. *J. Mach. Learn. Res.* **2011**, *12*, 2825–2830.

(51) Pedregosa, F.; Varoquaux, G.; Gramfort, A.; Michel, V.; Thirion, B.; Grisel, O.; Blondel, M.; Prettenhofer, P.; Weiss, R.; Dubourg, V.; Vanderplas, J.; Passos, A.; Cournapeau, D.; Brucher, M.; Perrot, M.; Duchesnay, E. Scikit-learn: Machine Learning in Python. *J. Mach. Learn. Res.* **2011**, *12*, 2825–2830.

(52) Freund, Y.; Schapire, R. E. A Decision-Theoretic Generalization of On-Line Learning and an Application to Boosting. *J. Comput. Syst. Sci.* **1997**, *55*, 119–139.

(53) Prokhorenkova, L.; Gusev, G.; Vorobev, A.; Dorogush, A. V.; Gulin, A. CatBoost: unbiased boosting with categorical features. *Proceedings of the 32nd NeurIPS* **2018**, 6639–6649.

(54) Joseph, V. R. Optimal Ratio for Data Splitting. *Stat. Anal.* **2022**, *15*, 531–538.

(55) Kaneko, H. Interpretation of Machine Learning Models for Data Sets with Many Features Using Feature Importance. *ACS Omega* **2023**, *8*, 23218–23225.

(56) Altmann, A.; Toloși, L.; Sander, O.; Lengauer, T. Permutation importance: a corrected feature importance measure. *Bioinformatics* **2010**, *26*, 1340–1347.

(57) Virtanen, P.; Gommers, R.; Oliphant, T. E.; et al. SciPy 1.0: Fundamental Algorithms for Scientific Computing in Python. *Nat. Methods* **2020**, *17* (3), 261–272.

(58) Claridge, T. D. W. *High-Resolution NMR Techniques in Organic Chemistry*; Elsevier: Oxford, **2016**.

(59) Riley, K. E.; Op’t Holt, B. T.; Merz, K. M., Assessment of density functional theory methods for the computation of heats of formation and ionization potentials of systems containing third row transition metals; *J. Phys. Chem. A* **2007**, *111* (38), 7433–744.

(60) Goerigk, L.; Grimme, S. Thermochemistry, Kinetics, and Noncovalent Interactions*;* J. *Chem. Theory Comput.* **2011**, *7*, 291–309.

(61) Parr, R. G.; Yang, W. *Density-Functional Theory of Atoms and Molecules*; Oxford University Press, **1989**.

(62) Barone, V. ; Adamo C.; Proton transfer in the ground and lowest excited states of malonaldehyde: A comparative density functional and post-Hartree–Fock study; *J. Chem. Phys*. **1996**, *105*, 11007–11019.

(63) Nakata, M.; Maeda, T. The Electronic Structures of 86 Million Molecules using B3LYP/6-31G Calculations*. *arXiv* **2023**, 2305.18454.
